# Prediction of transition to psychosis from an at-risk mental state using structural neuroimaging, genetic, and environmental data

**DOI:** 10.3389/fpsyt.2022.1086038

**Published:** 2023-01-19

**Authors:** Vânia Tavares, Evangelos Vassos, Andre Marquand, James Stone, Isabel Valli, Gareth J. Barker, Hugo Ferreira, Diana Prata

**Affiliations:** ^1^Instituto de Biofísica e Engenharia Biomédica, Faculdade de Ciências, Universidade de Lisboa, Lisbon, Portugal; ^2^Faculdade de Medicina, Universidade de Lisboa, Lisbon, Portugal; ^3^Social, Genetic and Developmental Psychiatry Centre, Institute of Psychiatry, Psychology and Neuroscience, King’s College London, London, United Kingdom; ^4^National Institute for Health Research Maudsley Biomedical Research Centre, South London and Maudsley National Health System Trust, London, United Kingdom; ^5^Donders Centre for Cognitive Neuroimaging, Donders Institute for Brain, Cognition and Behaviour, Radboud University, Nijmegen, Netherlands; ^6^Department of Cognitive Neuroscience, Radboud University Medical Centre, Nijmegen, Netherlands; ^7^Brighton and Sussex Medical School, University of Sussex, Brighton, United Kingdom; ^8^Department of Psychosis Studies, Institute of Psychiatry, Psychology and Neuroscience, King’s College London, London, United Kingdom; ^9^Institut d’Investigacions Biomèdiques August Pi i Sunyer, University of Barcelona, Barcelona, Spain; ^10^Department of Neuroimaging, Institute of Psychiatry, Psychology and Neuroscience, King’s College London, London, United Kingdom; ^11^Department of Old Age Psychiatry, Institute of Psychiatry, Psychology and Neuroscience, King’s College London, London, United Kingdom

**Keywords:** machine learning, biomarker, schizophrenia, ARMS, prognosis

## Abstract

**Introduction:**

Psychosis is usually preceded by a prodromal phase in which patients are clinically identified as being at in an “At Risk Mental State” (ARMS). A few studies have demonstrated the feasibility of predicting psychosis transition from an ARMS using structural magnetic resonance imaging (sMRI) data and machine learning (ML) methods. However, the reliability of these findings is unclear due to possible sampling bias. Moreover, the value of genetic and environmental data in predicting transition to psychosis from an ARMS is yet to be explored.

**Methods:**

In this study we aimed to predict transition to psychosis from an ARMS using a combination of ML, sMRI, genome-wide genotypes, and environmental risk factors as predictors, in a sample drawn from a pool of 246 ARMS subjects (60 of whom later transitioned to psychosis). First, the modality-specific values in predicting transition to psychosis were evaluated using several: (a) feature types; (b) feature manipulation strategies; (c) ML algorithms; (d) cross-validation strategies, as well as sample balancing and bootstrapping. Subsequently, the modalities whose at least 60% of the classification models showed an balanced accuracy (BAC) statistically better than chance level were included in a multimodal classification model.

**Results and discussion:**

Results showed that none of the modalities alone, i.e., neuroimaging, genetic or environmental data, could predict psychosis from an ARMS statistically better than chance and, as such, no multimodal classification model was trained/tested. These results suggest that the value of structural MRI data and genome-wide genotypes in predicting psychosis from an ARMS, which has been fostered by previous evidence, should be reconsidered.

## 1. Introduction

Psychosis is a severe condition usually within the context of a mental disorder such as a schizophrenia, some neurological disorders (e.g., Alzheimer’s disease) or other medical conditions (e.g., induced by drugs or illicit substances), characterized by a disconnection from reality (1). The onset of psychosis, when in the context of a mental disorder, is typically preceded by a prodromal phase that lasts months to years (2); and usually starts early during adolescence and precedes the onset of psychotic symptoms by 10 or more years (3). In this prodromal phase, subtle and subjectively experienced disturbances in mental processes emerge (basic symptoms). These are the first manifestations of the neurobiological processes underlying psychosis and are mainly distinguished from other symptoms (i.e., positive or negative symptoms) by their self-experience nature (4). As the course of the psychotic illness evolves, increasingly disabling behavioral symptoms start to emerge, generally called negative symptoms, in particular a reduction of motivation and/or expressiveness (5). Additionally, cognitive deficits in attention, memory, reasoning, lack of concentration and executive functioning appear (6). Lastly, positive symptoms emerge, such as hallucinations, delusions, disorganized speech, and behavior (1).

A patient may be clinically identified as being at a late prodromal phase of psychosis or having an “At Risk Mental State” (hereinafter: ARMS) if they present a functional decline in association with one or more of the following commonly used criteria (2, 7): (1) attenuated psychotic symptoms (APS), such as delusions, hallucinations, or disorganized speech with a frequency of at least once per week in the past month; (2) a brief limited intermittent psychotic (BLIP) episode lasting less than 1 week which resolves without antipsychotic medication; or (3) a genetic liability to psychosis or schizotypal traits, i.e., having either a first-degree relative with psychosis or a schizotypal personality disorder.

Transition to psychosis from an ARMS may be evaluated based on the severity, frequency, and total duration of the psychotic symptoms, i.e., when the subject experiences a first episode of psychosis (FEP). Subjects with an ARMS and seeking help have a transition rate to psychosis of about 9% in the first 6 months and 25% in the first 3 years (8) and, in particular, an increased risk of transition to schizophrenia of 15.7% within an average period of 2.35 years, as shown by a meta-analysis (9). Thus, most of the people with an ARMS who later develop a psychotic illness will be diagnosed with schizophrenia. Furthermore, since about 70% of subjects diagnosed with an ARMS never develop a full-blown psychotic illness (9), these people may benefit from a less intensive treatment to ameliorate symptoms or need no treatment at all. Such increase in treatment cost-effectiveness would represent a substantial decrease in healthcare costs, and treatment burden to patients, including pharmacological side effects. However, there is no method for distinguishing between individuals with an ARMS who will subsequently develop a psychotic illness from those who will not (i.e., before a FEP onset).

Given the above need, an effective, precise, and quantitative tool for the prediction of transition to psychosis from an ARMS has been sought by several studies employing machine learning (ML) methods and structural magnetic resonance imaging (sMRI). Indeed, several studies have consistently showed prediction of transition to psychosis from as ARMS with accuracies ranging between 74 and 84% (10–15). Transition to psychosis from an ARMS using only sMRI and ML was first predicted using whole-brain gray matter volume metrics with an accuracy of 82% [(15 ARMS who transitioned to psychosis (ARMS-T) and 18 who did not (ARMS-NT)] (10). This finding was later replicated: (a) in an independent sample by the same group [balanced accuracy (BAC) = 84%, 16 ARMS-T and 21 ARMS-NT] (11); (b) combining both these samples (BAC = 80%, 33 ARMS-T and 33 AMRS-NT) (12); (c) using also one of the above samples for graph-extracted network metrics from cortical gyrification (BAC = 81%, 16 ARMS-T and 63 ARMS-NT) (15), and regional gray matter metrics (BAC = 74%, 16 ARMS-T and 19 ARMS-NT) (14); and (d) using regional gray matter metrics in an independent sample (BAC = 77%, 17 ARMS-T and 17 ARMS-NT; specificity of a replication sample of individuals with an ARMS who did not develop psychosis = 68%, 40 ARMS-NT) (13). To date, only two, relatively small, ARMS samples have been used for sMRI and ML analysis: FETZ (10, 12, 15) and FePsy (11, 12, 14). Thus, the robustness and generalizability of the above findings are still unclear due to possible specific sample characteristics, i.e., small sample sizes (from 33 individuals to at most 79 individuals with ARMS), with several studies stemming from a single site (10, 11, 13–15) or a combination of previously studied sites (12), which makes them not actual replications, with one exception (13).

Interestingly, to the best of our knowledge, genetic data has been explored for the prediction of the transition to psychosis from an ARMS only once (16). In this study, a schizophrenia polygenic risk score (PRS) was able to predict transition to psychosis in individuals with an European [area under the curve (AUC) = 0.65; 32 ARMS-T and 92 ARMS-NT] and with a Non-European (AUC = 0.59; 48 ARMS-T and 156 ARMS-NT) ancestry, respectively. This is despite there being several classification studies showing that genetic markers can predict schizophrenia (17–22), FEP (23) or ARMS (23), both of individual polymorphisms (18, 19, 21, 23) or, composite polygenic scores (20–22), and gene expression profiles (24). From an environmental exposure perspective, and to the best of our knowledge, environmental data have never been explored for predicting individual transition to psychosis from an ARMS.

The combination of neuroimaging measures and genetics or environmental measures, using ML, has, to the best of our knowledge, been explored once to predict ARMS prognosis (i.e., transition to psychosis from an ARMS) in a study running in parallel to ours (25). Therein, a large sample from the PRONIA project (26 ARMS-T and 308 ARMS-NT from 7 sites) was used to build a sequential stacked multimodal model using clinical-neurocognitive (including environmental data), genetic (in the form of a PRS for schizophrenia) and neuroimaging (in the form of voxel-based gray matter volume maps) data and - unlike the present study–human prognostic ratings, showing a final balanced accuracy in predicting transition to psychosis of 86%.

In the present longitudinal prognostic biomarker study, we aimed to explore the use of ML models trained with sMRI, genetic, and environmental baseline data to predict the individual-level transition to psychosis from an ARMS within a 2-year follow up. While providing such preliminary (given the unprecedented data combinations/features and a limited sample size) evidence at the multimodal level, we took the opportunity to attempt to replicate previous promising sMRI-ML findings of studies using similar or smaller sample size (10–15). Methods-wise, we used naturalistically diverse samples but balanced them for demographic (age and sex) and imaging (scan acquisition sMRI protocol) variables. We set out to train and test modality-specific models first and then, provided these performed above chance-level, a multimodal one. For the sMRI data, we used state-of-the-art preprocessing and ML pipelines; and explored several unprecedented combinations of brain structural measures, feature manipulation and cross-validation (CV) strategies. For the genetic data, we explored several approaches: a schizophrenia PRS (26), individual GWA-implicated SNPs (27), and a brain-specific expression Quantitative Trait Loci (eQTL) score. For the environmental data, we employed a schizophrenia environmental risk score (ERS) (28), and individual risk factors.

## 2. Materials and methods

### 2.1. Sample description

The total sample consisted of 246 individuals with an ARMS, recruited at first presentation from consecutive referrals to the Outreach and Support in South London (OASIS) high-risk service, South London and Maudsley NHS Foundation Trust (29). The presence of ARMS was assessed using the CAARMS, a detailed clinical assessment (30). When the subjects were first diagnosed as having an ARMS (i.e., baseline) a set of data were acquired: (a) a sMRI scan; (b) genome-wide genotypes; and (c) assessment of environmental risk exposures. Subjects were labeled as transitioned to psychosis (ARMS-T) if they later presented a FEP or as not-transitioned to psychosis (ARMS-NT) if they did not present a FEP within a period of at least 2 years. For a detailed description of the recruitment, inclusion and exclusion criteria please refer to the Supplementary material. Additional socio-demographic and clinical measures were also assessed at baseline, including: age; sex; handedness; self-reported ethnicity; full scale intelligence quotient measured by the National Adult Reading Test (31); years of education; and global assessment of function using the GAF instrument tool at baseline and at follow-up (32), and CAARMS (at baseline and follow-up) (30). Regarding the sMRI, genetic and environmental sub-samples: 99, 135 and all the 246 individuals with an ARMS had a baseline sMRI scan (Table 1), genome-wide genotyped data (Table 2), and environmental risk factors assessment data (Table 3), respectively (more details in the Supplementary material). Over the 2-years follow-up period, 23, 41, and 60 individuals at an ARMS from each of the previous sub-samples developed psychosis (AMRS-T) and the remaining 15, 94, and 186 did not (ARMS-NT), respectively. Moreover, part of the study’s data collection occurred under the Genetic and Psychosis (GAP) umbrella project (33). Ethics approval was obtained by the NHS South East London Research Ethics Committee (Project GAP; Ref. 047/04), consistent with the Helsinki Declaration of 1975 (as revised in 2008) and all subjects gave written informed consent.

**TABLE 1:** Socio-demographic and clinical information of the At Risk Mental State (ARMS) sample with structural MRI data.

	Protocol 1		Protocol 2		Protocol 3		Group comparison
	ARMS-T (*n* = 14)	ARMS-NT (*n* = 19)	ARMS-T (*n* = 3)	ARMS-NT (*n* = 16)	ARMS-T (*n* = 6)	ARMS-NT (*n* = 41)	
Age at baseline (years)	23.2 ± 3.4 [15.6 26.9]	24.5 ± 4.8 [19.2 34.5]	26.2 ± 7.0 [20.1 33.8]	24.5 ± 5.2 [17.8 35.3]	23.4 ± 4.5 [17.5 29.2]	21.8 ± 4.3 [17.1 33.1]	Protocol: *p* = 0.271 Transition: *p* = 0.592 Protocol × Transition: *p* = 0.447
Age at follow-up or transition (years)	25.6 ± 4.2 [17.3 33.4]	32.7 ± 5.2 [22.6 43.9]	29.2 ± 5.4 [20.2 38.8]	28.8 ± 5.6 [22.9 43.1]	25.2 ± 4.8 [18.3 31.0]	25.6 ± 4.8 [20.3 41.2]	Protocol: *p* = 0.027* Transition: *p* = 0.099 Protocol × Transition: *p* = 0.025*
Age at scan (years)	23.0 ± 3.6 [17.5 27.8]	23.9 ± 4.8 [18.5 34.8]	27.0 ± 8.2 [20.2 36.1]	25.1 ± 5.4 [18.6 37.4]	24.1 ± 4.8 [18.3 30.8]	22.4 ± 4.6 [17.7 38.3]	Protocol: *p* = 0.261 Transition: *p* = 0.499 Protocol × Transition: *p* = 0.541
Interval between baseline and scan age (years)	–0.2 ± 1.4 [–2.3 1.9]	–0.5 ± 1.1 [–2.3 2.1]	0.9 ± 1.3 [0.1 2.4]	0.5 ± 0.5 [0.1 2.1]	0.6 ± 0.5 [0.2 1.6]	0.6 ± 1.0 [0.0 5.1]	Protocol: *p* < 0.001*** Transition: *p* = 0.419 Protocol × Transition: *p* = 0.795
Sex (male/female)	11/3	9/10	2/1	14/2	3/3	19/22	Protocol × Transition: Protocol 1: *p* = 0.070 Protocol 2: *p* = 0.422 Protocol 3: *p* = 1
Handedness^a^ (right/left/ambidextrous)	12/0/1	16/0/0	3/0/0	13/1/0	4/0/0	36/4/0	Protocol × Transition: Protocol 1: *p* = 0.448 Protocol 2: *p* = 1 Protocol 3: *p* = 1
Self-reported ethnicity (White/Black/Asian/other)	7/5/1/1	11/5/1/2	2/1/0/0	13/1/1/1	4/1/1/0	19/19/1/2	Protocol × Transition: Protocol 1: *p* = 0.933 Protocol 2: *p* = 0.530 Protocol 3: *p* = 0.212
Years of education	13.4 ± 2.1 [10 18]	13.1 ± 1.9 [10 17]	11.7 ± 2.3 [9 13]	14.1 ± 2.6 [11 20]	15.2 ± 2.5 [11 18]	13.0 ± 2.2 [9 20]	Protocol: *p* = 0.298 Transition: *p* = 0.966 Protocol × Transition: *p* = 0.024*
IQ (z-standardized)^b^	–1.1 ± 1.1 [–2.1 1.0]	0.0 ± 1.1 [–2.1 1.8]	0.1 ± 0.1 [0.0 0.2]	0.5 ± 0.9 [–1.3 1.6]	−0.1 ± 1.3 [–2.1 1.6]	0.1 ± 1.1 [–2.1 3.5]	Protocol: *p* = 0.427 Transition: *p* = 0.252 Protocol × Transition: *p* = 0.923
GAF at baseline	52.9 ± 16.0 [35 90]	57.8 ± 11.4 [35 75]	58.7 ± 3.2 [55 61]	58.6 ± 9.9 [41 75]	50.3 ± 11.4 [35 65]	53.6 ± 14.8 [0 75]	Protocol: *p* = 0.402 Transition: *p* = 0.475 Protocol × Transition: *p* = 0.877
GAF at follow-up^c^	49.3 ± 18.6 [10 69]	58.5 ± 17.9 [20 94]	27.3 ± 6.8 [22 35]	62.3 ± 13.5 [46 93]	52.5 ± 20.0 [30 78]	66.2 ± 13.6 [33 87]	Protocol: *p* = 0.064 Transition: *p* < 0.001*** Protocol × Transition: *p* = 0.095
CAARMS at baseline^d^	33.2 ± 13.0 [9 56]	28.4 ± 15.3 [8 58]	29.3 ± 21.9 [12 54]	23.2 ± 14.3 [0 51]	39.7 ± 24.1 [0 69]	28.5 ± 16.7 [0 81]	Protocol: *p* = 0.505 Transition: *p* = 0.153 Protocol × Transition: *p* = 0.824
CAARMS at follow-up^e^	19.6 ± 23.0 [0 63]	11.6 ± 10.9 [0 31]	42.0 ± 43.3 [6 90]	14.7 ± 18.4 [0 54]	42.7 ± 42.1 [0 102]	15.5 ± 17.2 [0 60]	Protocol: *p* = 0.082 Transition: *p* = 0.001*** Protocol × Transition: *p* = 0.262

Data format: mean ± standard deviation [min max]. Information not available for ^a^1 ARMS-T and 3 ARMS-NT (Protocol 1), 2 ARMS-NT (Protocol 2), 2 ARMS-T and 1 ARMS-NT (Protocol 3); ^b^1 ARMS-T and 1 ARMS-NT (Protocol 2), 1 ARMS-NT (Protocol 3); ^c^2 ARMS and 5 ARMS-NT (Protocol 1), 4 ARMS-NT (Protocol 2), 8 ARMS-NT (Protocol 3); ^d^2 ARMS-T and 7 ARMS-NT (Protocol 1), 3 ARMS-NT (Protocol 2), 2 ARMS-NT (Protocol 3); ^e^3 ARMS-T and 6 ARMS-NT (Protocol 1), 3 ARMS-NT (Protocol 2), 8 ARMS-NT (Protocol 3). ARMS, at-risk mental state; ARMS-T, individuals at ARMS that did not transition to psychosis; ARMS-NT, individuals at ARMS that did not transitioned to psychosis. *p < 0.05; ***p < 0.001.

**TABLE 2:** Socio-demographic and clinical information of the At Risk Mental State (ARMS) sample with genetic data and an European ancestry.

	ARMS-T (*n* = 21)	ARMS-NT (*n* = 54)	Group comparison
Age at baseline (years)	23.8 ± 5.3 [15.6 33.8]	22.5 ± 4.0 [14.6 34.5]	*p* = 0.284
Age at follow-up or transition (years)	25.3 ± 5.9 [17.3 38.8]	27.9 ± 5.1 [18.5 43.9]	*p* = 0.069
Sex (male/female)	14/7	30/24	*p* = 0.380
Years of education	13.0 ± 2.2 [10.0 18.0]	12.0 ± 4.4 [0 18.0]	*p* = 0.292
IQ (z-standardized)^a^	0.1 ± 1.0 [–2.1 2.2]	0.2 ± 1.0 [–2.1 1.8]	*p* = 0.678
GAF at baseline	54.0 ± 15.7 [0 80]	53.6 ± 16.0 [0 78]	*p* = 0.923
GAF at follow-up^b^	47.8 ± 24.3 [0 79]	59.2 ± 21.0 [0 94]	*p* = 0.050
CAARMS at baseline^c^	37.6 ± 17.5 [6 69]	29.9 ± 16.2 [0 81]	*p* = 0.097
CAARMS at follow-up^d^	24.4 ± 27.9 [0 90]	12.4 ± 14.0 [0 60]	*p* = 0.019*

Data format: mean ± standard deviation [min max]. Information not available for ^a^2 ARMS-T and 9 ARMS-NT; ^b^4 ARMS-NT; ^c^1 ARMS and 9 ARMS-NT; ^d^1 ARMS-T and 3 ARMS-NT. ARMS, at-risk mental state; ARMS-T, individuals at ARMS that did not transition to psychosis; ARMS-NT, individuals at ARMS that did not transitioned to psychosis. *p < 0.05.

**TABLE 3:** Socio-demographic and clinical information of the At Risk Mental State (ARMS) sample with environmental data (with less than 20% of the environmental risk factors missing).

	ARMS-T (*n* = 37)	ARMS-NT (*n* = 97)	Group comparison
Age at baseline (years)	23.6 ± 4.8 [15.6 33.6]	21.9 ± 3.7 [14.6 33.1]	*p* = 0.027*
Age at follow-up or transition (years)^a^	25.6 ± 5.6 [17.3 39.2]	27.1 ± 4.7 [18.5 41.2]	*p* = 0.131
Sex (male/female)	22/15	50/47	*p* = 0.411
Years of education^b^	13.2 ± 2.7 [8 18]	13.3 ± 2.0 [9 18]	*p* = 0.686
IQ (z-standardized)^c^	−0.3 ± 1.0 [–2.1 2.2]	0.1 ± 1.0 [–2.1 3.5]	*p* = 0.049*
GAF at baseline^d^	55 ± 12.5 [35 90]	56.7 ± 8.6 [40 80]	*p* = 0.523
GAF at follow-up^e^	50.4 ± 19.9 [10 88]	63.2 ± 14.2 [20 94]	*p* =< 0.001*
CAARMS at baseline^f^	30.9 ± 19.4 [0 78]	28.3 ± 16.0 [0 81]	*p* = 0.478
CAARMS at follow-up^g^	29.7 ± 31.2 [0 102]	13.3 ± 14.2 [0 60]	*p* =<0.001*

Data format: mean ± standard deviation [min max]. Information not available for ^a^1 ARMS-T; ^b^5 ARMS-T and 6 ARMS-NT; ^c^7 ARMS-T and 13 ARMS-NT; ^d^5 ARMS-T and 4 ARMS-NT; ^e^5 ARMS-T and 8 ARMS-NT; ^f^6 ARMS-T and 13 ARMS-NT; ^g^4 ARMS-T and 8 ARMS-NT; subject. ARMS, at-risk mental state; ARMS-T, individuals at ARMS that did not transition to psychosis; ARMS-NT, individuals at ARMS that did not transition to psychosis. *p < 0.05.

Socio-demographic and clinical variables were analyzed using a two-tailed independent *t*-test or a Univariate Analysis of Variance (ANOVA) for continuous data and a chi-square test or Fisher’s exact test (if there were less than 5 subjects in one group) for ordinal data (Tables 1–3). These statistical analyses were performed using the Statistical Package for the Social Sciences 26 (SPSS 26 for Windows, Chicago, IL, USA).

### 2.2. Structural neuroimaging data

#### 2.2.1. Structural magnetic resonance imaging acquisition

Structural magnetic resonance imaging (sMRI) scans were acquired with one of two scanners (one with a field strength of 1.5T, the other 3T) using one of three 3-Dimensional enhanced fast gradient echo protocols (detailed description in Supplementary material).

#### 2.2.2. Image processing

High spatial resolution volumetric T1-weighted images were processed with the Computational Anatomy Toolbox for Statistical Parametric Mapping (SPM) –12 (CAT12; v1092^1^), an SPM12 add-on (v6909^2^) using default settings and MATLAB (9.3) as we have described elsewhere (34) (detailed description in Supplementary material). In summary, gray and white matter volumes for 64 regions-of-interest (ROIs; description of each ROI is in the Supplementary Table 1) were extracted using the Hammers atlas (35). Additionally, regional-based cortical thickness and surface measures (i.e., folding measures)–gyrification index, the depth of sulci and the measurement of local surface complexity were extracted for 68 ROIs (description of each ROI is in the Supplementary Table 2) defined by the Desikan–Killiany atlas (36).

#### 2.2.3. Image quality control

The quality of each processed image was empirically assessed using the quality assurance framework of CAT12 (detailed description in the Supplementary material). We set the subject’s image inclusion threshold at D (sufficient), i.e., only subjects whose images had an image quality rate of A (excellent) to D (sufficient) (in a scale that goes up to F–unacceptable/failed) were included in the final sample, as it has been shown that typical scientific (clinical) data get good-to-satisfactory ratings (37). All this study’s images passed the above criteria and thus were included in all analyses (see Supplementary material for more details).

### 2.3. Genetic data

Genotyping procedures have been previously described (26, 38). In summary, samples were genotyped at two different sites with two distinct chips (Illumina HumanCore Exome BeadChip and Genome-wide Human SNP Array 6.0). A standard quality control screening (exclusion of SNPs with low minor allele frequency, high genotypic failure and not in Hardy Weinberg equilibrium) followed by imputation procedures were conducted. Then, samples from both sites were merged by keeping only the overlapped imputed SNPs followed by a second quality control screening. Finally, a population stratification analysis was conducted with principal component analysis (PCA) to select only subjects with a European ancestry (the number of subjects per self-reported ethnicity is in the Supplementary Table 3). For a detailed description see the Supplementary material.

### 2.4. Environmental data

Each subject was assessed on at least one of eight environmental risk factors: (1) tobacco and (2) cannabis consumption; (3) being migrant; (4) belonging to an ethnic minority; (5) the upbringing urbanicity level; (6) the parental age at birth; (7) the presence of childhood trauma; and (8) the season of birth (detailed description of how the risk for psychosis was assessed in each factor is in Supplementary material).

### 2.5. Machine learning approach

**FIGURE 1:**
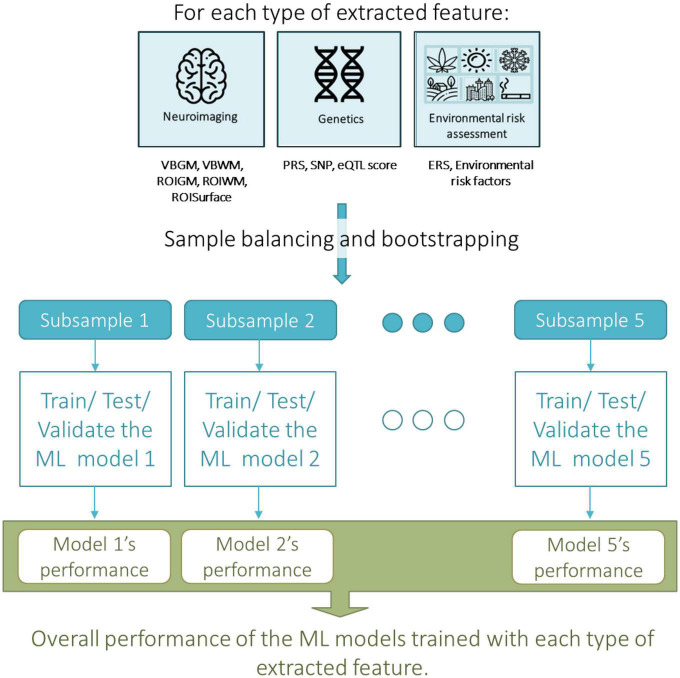
Overall machine learning approach taken for assessing the predictive value, i.e., the accuracy, of each type of extracted neuroimaging, genetic or environmental feature in predicting transition to psychosis from an At Risk Mental State (ARMS). ERS, environmental risk score; eQTL score, expression quantitative trait loci; PRS, polygenic risk score; ROIGM, regional-based gray matter volumes; ROISurface, surface-based regional cortical thickness, and gyrification, sulci, and complexity indexes; ROIWM, regional-based white matter volumes; SNP, single nucleotide polymorphism; VMGM, voxel-based gray matter volume maps; VMWM, voxel-based white matter volume maps.

**FIGURE 2:**
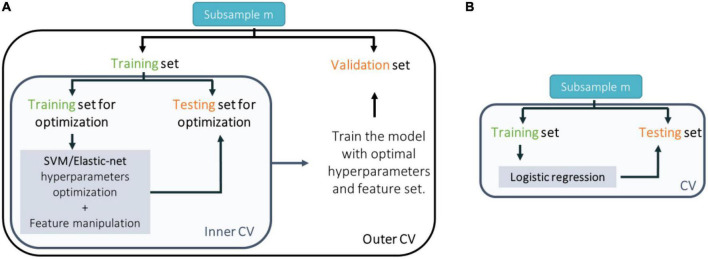
Scheme of the cross-validation (CV) approach taken to train, test, and validate classification models trained with **(A)** neuroimaging data and support vector machines (SVM); genetic (single nucleotide polymorphisms or expression quantitative trait loci) or environmental (environmental risk factors) data and elastic-net; or **(B)** genetic (polygenic risk score) or environmental (environmental risk score) data and logistic regression.

Several ML strategies to generate prediction models for transition to psychosis from sMRI data using our ARMS sample were investigated (Figures 1, 2). These include: (a) sample balancing and bootstrapping; and testing several: (b) feature types; (c) feature manipulation approaches; and (d) CV approaches. The analyses were conducted using the neuroimaging ML tool NeuroMiner v1.0 ELESSAR^3^ for sMRI data, chosen given that it was used in the previous above-mentioned ARMS prognosis studies and provided therein high accuracy results (12, 39, 40), and R software 4.0.5 (41) for genetic (16) and environmental data. As detailed below, we have used SVM on the neuroimaging data since that is the approach which not only is more often employed with sMRI data but also that which has shown higher accuracies in psychiatric diagnostic classifications using sMRI data including in the ARMS population (10–14) which we herein attempt to replicate. We have used elastic-net algorithm for the genetic data (SNPs and eQTL scores) and environmental risk factors as it a well-suited method for dealing with high-dimensional data and possibly correlated data; and it performs an embedded feature selection and model fitting at once. The PRS and the environmental risk score were analyzed with logistic regression, given that only one feature was used.

#### 2.5.1. Sample balancing and bootstrapping

The final sample used in the ML analyses was defined by all the ARMS-T subjects available (23 subjects for the sMRI predictors, 19 for the PRS predictor, 21 for the SNP’s alleles predictors, 21 for eQTL scores predictors, 37 for the ERS predictor, and 17 for the individual environmental predictors), and the same number of ARMS-NT subjects randomly selected to match the ARMS-T for age and sex (for each data modality), and for scan acquisition protocol (for sMRI data). The matching criteria for age and sex were based on the non-rejection of the null hypothesis (i.e., *p* > 0.05) that the ARMS-T and ARMS-NT groups had the same median age (tested with a two-sided Mann–Whitney *U*-test) and sex proportions (tested with a two-sided chi-square statistic). The matching for the scan acquisition protocol was done in a one-to-one manner, i.e., the number of ARMS-NT subjects within each protocol is the same as the number of ARMS-T. Of note, we have considered the approach of applying a class-weighted support vector machine for our neuroimaging measures and have detected that differences in terms of accuracies between a model with weights vs. no-weights (considering the full unbalanced samples) were practically null (results not shown)–and therefore we did not pursue that approach. Then, each subsampling was repeated five times, i.e., 5 bootstrapped samples were created, and the subsequent ML analyses were conducted for each of the bootstrapped sample.

#### 2.5.2. Feature types

##### 2.5.2.1. Structural magnetic resonance imaging data

Individual ML models were trained and validated for each of the following brain measures: (a) voxel-based gray matter (VBGM) maps (297,811 initial features); (b) voxel-based white matter (VBWM) maps (204,706 initial features); (c) regional-based gray (ROIGM) and (d) white (ROIWM) matter volumes (each with 64 initial features) scaled to the total intracranial volume (TIV); and (e) surface-based regional cortical thickness, and gyrification, sulci, and complexity indexes (ROISurface; 272 initial features). Each feature is scaled between 0 and 1 before entering a support vector machine (SVM) classification algorithm.

##### 2.5.2.2. Genetic data

We tested whether a PRS which we have previously found to predict (*R*^2^= 0.94) a cross-sectional diagnosis of FEP (vs. healthy controls) would be a good longitudinal predictor for ARMS prognosis. Following the same methodology (26), this PRS was computed as the sum of SNPs alleles statistically associated with schizophrenia in a GWAS meta-analysis study (42) weighted by the effect size of that association (more details in Supplementary material). In addition, two other novel prediction models using the present ARMS sample were trained and tested. One used SNPs’ alleles (79,247 SNPs) as predictors and the other used eQTL scores of genes expressed in brain tissue (141 genes across several brain tissues). Both SNPs and genes’ eQTL scores were chosen as the ones most associated with psychosis as ascertained in a recent meta-analysis (27). The eQTL score of each gene was extracted with the eGenScore which we developed and published previously (43) and it was computed as the sum of the alleles of SNPs showing a statistically significant association with the brain gene expression in a standard genomic and transcriptomic sample weighted by the size of that effect (further details available in Supplementary material).

##### 2.5.2.3. Environmental data

We tested whether an ERS for psychosis which we have previously developed (28) would be a good longitudinal predictor for ARMS prognosis. Only subjects with less than 20% of missing information (i.e., missing data for less than 2 environmental risk factors) were considered for the ERS-based ML analysis. Therefore, the final sample included 37 ARMS-T subjects and 97 ARMs-NT subjects. Then, each environmental risk factor (see Section “2.4. Environmental data”) was used as an individual feature in the model. For this ML analysis only subjects with information for all the environmental risk factors (i.e., with no missing information) were considered (i.e., 17 ARMS-T and 49 ARMS-NT subjects). Further details available in Supplementary material.

#### 2.5.3. Feature manipulation

Feature manipulation was performed only in ML analyses using sMRI data. In particular, feature dimensionality reduction was performed for VBGM and VBWM features using robust PCA (44, 45). Here the robust PCA was applied during the inner CV cycle (see Section “2.5.5. Cross-validation”). The number of principal components that were retained explained up to 80% of the variance in the data and were limited by the inner CV cycle’s sample size, *n*, i.e., a maximum of only *n*/2 components could indeed be extracted. Supplementary Table 5 shows the maximum number of principal components that can be extracted for each inner CV cycle in each CV scheme that was used (see also Section “2.5.5. Cross-validation”) (for detailed description see the Supplementary material).

Feature selection was performed on regional brain features (i.e., ROIGM, ROIWM, and ROISurface) using a greedy forward search feature selection algorithm. This is a stepwise algorithm that starts with an empty set of features and then tests the predictive value of every single feature, selecting the ones improving the overall accuracy across the inner CV cycle folds (see Section “2.5.5. Cross-validation”). The final set of features is, then, composed by the 10% most predictive variables. Additionally, no feature selection, i.e., using the total number regional brain features, was also tested.

#### 2.5.4. Machine learning algorithm

Binary classification of transition to psychosis from an ARMS (i.e., ARMS-T vs. ARMS-NT) was performed using linear SVM for sMRI data, and logistic regression and elastic net for both genetic and environmental data.

##### 2.5.4.1. Support vector machine classification

Binary classification of transition to psychosis from an ARMS (i.e., ARMS-T vs. ARMS-NT) using sMRI data was performed using linear SVM (46, 47). In this study we exclusively used a linear kernel SVM to reduce the risk of overfitting the data (given our final sample size being relatively small). Furthermore, the linear SVM classifier has a penalty parameter C that controls the trade-off between having zero training error and allowing misclassification. Herein, a parameter search was carried out to identify the optimal C value (i.e., 2^*l*^,*l*[−5:1:4]) in the inner CV cycle (see Section “2.5.5. Cross-validation”).

##### 2.5.4.2. Logistic regression for classification

Binary classification of transition to psychosis from an ARMS (i.e., ARMS-T vs. ARMS-NT) using genetic (PRS) or environmental (ERS) data was performed using simple logistic regression. A threshold of 0.5 was applied to the probability of observing the outcome, i.e., an ARMS-T (see Supplementary material for more details).

##### 2.5.4.3. Elastic net for classification

Binary classification of transition to psychosis from an ARMS (i.e., ARMS-T vs. ARMS-NT) using genetic (psychosis-associated SNPs or eQTL scores of psychosis-associated genes) or environmental (environmental risk factors) data was performed using logistic regularized regression with elastic net (48) using hyperparameters search to identify the optimal *l_1_* and λ values (regression weights shrinkage) (i.e., *l*_1_0:0.1:1;λ0.01:0.01:1) in the inner CV cycle (see Section “2.5.5. Cross-validation”) (for detailed description see the Supplementary material). The elastic net was implemented using the “glmnet” v4.0 R package.

#### 2.5.5. Cross-validation

Each model (trained with sMRI, psychosis-associated SNPs or eQTL scores of psychosis-associated genes and environmental risk factors) was trained in a nested-CV scheme for hyperparameter tuning (in the inner CV cycle) and to estimate the generalizability of the trained prediction model and its performance (in the outer CV cycle) (Figure 2A). For more details see the Supplementary material. For sMRI models, we tested three different nested-CV schemes: (a) leave-one scan acquisition protocol-out (LSO); (b) leave-one per group from the same scan acquisition protocol-out (LPO); and (c) classic 5-fold CV. For the remaining sMRI, genetic (trained with psychosis-associated SNPs or eQTL scores of psychosis-associated genes data) and environmental (trained with environmental risk factors data) models, nested-CV was defined with an inner 5-fold and an outer leave-one per group-out (LPO) CV schemes. Furthermore, the logistic regression (trained with genetic–PRS–and environmental–ERS–data) was trained and tested in a simple LPO CV scheme (Figure 2B).

#### 2.5.6. Performance measures

Each model’s performance was evaluated using measures derived from the confusion matrix: sensitivity; specificity; BAC; positive likelihood ratio; negative likelihood ratio; and diagnostic odds ratio (DOR). Moreover, permutation testing was used to test if the BAC was higher than chance–50%–with a statistical significance of 5% (For a detailed description of each measure see the Supplementary material).

The prediction ability of each tested combination of feature type, feature manipulation, and CV scheme was defined as significant if the BAC was higher than chance–50% in at least 3 out of 5 bootstrapped samples. evaluated by testing the statistical significance of the median BAC across bootstrapped samples using a one-tailed Wilcoxon signed rank test (i.e., to test if the median BAC across bootstrapped samples is higher than chance– 50%, with a statistical significance level of 5%). *P*-values were not adjusted for multiple comparisons due to non-independence of the samples used in each statistical test.

## 3. Results

**FIGURE 3:**
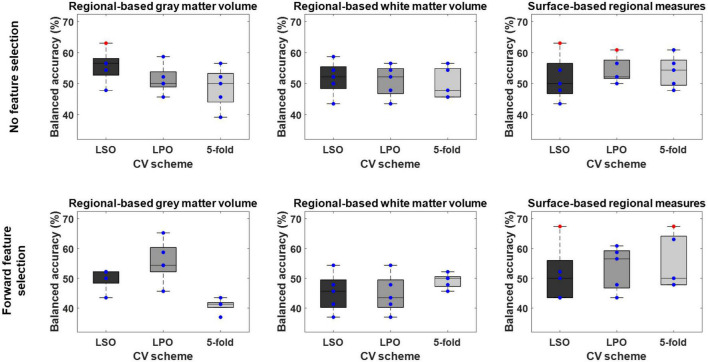
Balanced accuracy across bootstrapped samples for each tested combination of regional feature type [i.e., regional-based gray and white matter volume; and surface-based regional cortical thickness, gyrification, sulci, and complexity indexes (surface-based regional measures)], feature selection [i.e., no feature selection; and forward feature selection (FFS)], and cross-validation (CV) scheme [i.e., leave-one scan acquisition protocol-out (LSO) CV; leave-one per group-out (LPO) CV; and 5-fold CV]. Dots represent the balanced accuracy value in each of the five bootstrapped samples and are red colored if the balanced accuracy is statistically significant (i.e., *p* < 0.05) or blue colored if it is not (i.e., *p* > 0.05). The statistical significance of the balanced accuracy in each bootstrapped sample was evaluated through permutation testing.

**FIGURE 4:**
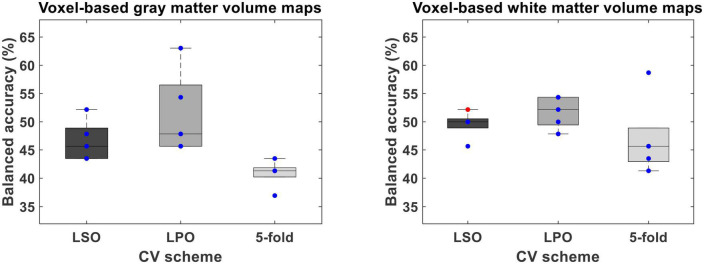
Balanced accuracy across bootstrapped samples for each tested combination of voxel-wise feature type [i.e., voxel-based gray (VBGM) and white (VBWM) matter volume maps], feature dimensionality reduction through principal component analysis and cross-validation (CV) scheme [i.e., leave-one scan acquisition protocol-out (LSO) CV; leave-one per group-out (LPO) CV; and **5-**fold CV. Dots represent the balanced accuracy value in each of the five bootstrapped samples and are red colored if the balanced accuracy is statistically significant (i.e., *p* < 0.05) or blue colored if it is not (i.e., *p* > 0.05). The statistical significance of the balanced accuracy in each bootstrapped sample was evaluated through permutation testing.

**FIGURE 5:**
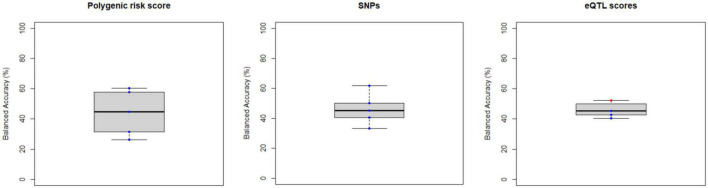
Balanced accuracy across bootstrapped samples for each model trained with the polygenic risk score, the list of psychosis-associated single nucleotide polymorphism (SNPs) or with the list of psychosis-associated genes for which an expression quantitative trait loci (eQTL) score was extracted. Dots represent the balanced accuracy value in each of the 5 bootstrapped samples and are red colored if the balanced accuracy is statistically significant (i.e., *p* < 0.05) or blue colored if it is not (i.e., *p* > 0.05). The statistical significance of the balanced accuracy in each bootstrapped sample was evaluated through permutation testing.

**FIGURE 6:**
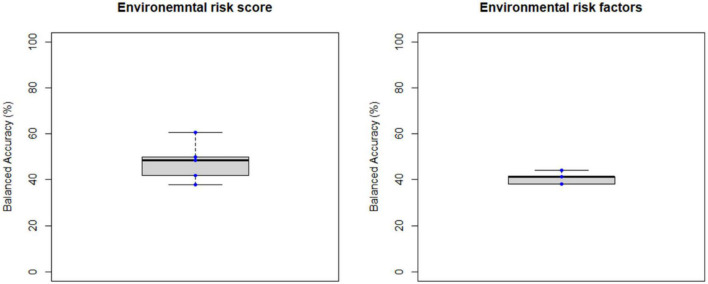
Balanced accuracy across bootstrapped samples for each model trained with the environmental risk score or with each environmental risk factors as features. Dots represent the balanced accuracy value in each of the 5 bootstrapped samples and are red colored if the balanced accuracy is statistically significant (i.e., *p* < 0.05) or blue colored if it is not (i.e., *p* > 0.05). The statistical significance of the balanced accuracy in each bootstrapped sample was evaluated through permutation testing.

Overall, the BAC of the classification models trained and validated using each combination of feature type (i.e., ROIGM, ROIWM, ROISurface, VBGM, or VBWM–for sMRI data; PRS, psychosis-associated SNPs or psychosis-associated brain eQTL score genes scores–for genetic data; or ERS or individual environmental risk factors–for environmental data), feature manipulation (i.e., feature dimensionality reduction through PCA; no feature selection; or forward feature selection), CV scheme (i.e., LSO CV; LPO CV; or 5-fold CV), and bootstrapped sample (i.e., one of the 5 samples) ranged from 37 to 67% for the classification models trained with sMRI (Tables 4, 5 and Figures 3, 4), from 26 to 62% for the models trained with genetic data (Table 6 and Figure 5) and from 38 to 61% for models trained with environmental data (Table 6 and Figure 6). The prediction ability of each model was not significant as less than 3 bootstrapped samples per each feature type showed a BAC statistically higher than chance–50%.

**TABLE 4:** Performance measures of each structural magnetic resonance imaging (sMRI) classification model based on brain regional features across bootstrapped samples.

	ROIGM		ROIWM		ROISurface	
	No-FS	FFS	No-FS	FFS	No-FS	FFS
**LSO CV scheme**						
SE (%)	55.7 ± 6.4 [47.8, 65.2]	59.1 ± 11.7 [47.8, 78.3]	57.4 ± 19.1 [30.4, 82.6]	62.6 ± 13.3 [52.2, 82.6]	41.7 ± 14.6 [26.1, 60.9]	39.1 ± 20.6 [17.4, 65.2]
SP (%)	55.7 ± 10.4 [43.5, 69.6]	40.9 ± 10.5 [26.1, 52.2]	46.1 ± 14 [21.7, 56.5]	27.8 ± 22.3 [0.0, 56.5]	61.7 ± 17.8 [34.8, 82.6]	63.5 ± 12.1 [43.5, 73.9]
BAC (%)	55.7 ± 5.5 [47.8, 63.0]	50.0 ± 3.8 [43.5, 52.2]	51.7 ± 5.6 [43.5, 58.7]	45.2 ± 6.6 [37.0, 54.3]	51.7 ± 7.4 [43.5, 63.0]	51.3 ± 9.8 [43.5, 67.4]
PLR	1.3 ± 0.3 [0.9, 1.9]	1.0 ± 0.1 [0.8, 1.1]	1.1 ± 0.2 [0.7, 1.4]	0.9 ± 0.2 [0.7, 1.2]	1.2 ± 0.4 [0.8, 1.8]	1.1 ± 0.6 [0.6, 2.1]
NLR	0.8 ± 0.2 [0.6, 1.1]	1.0 ± 0.2 [0.8, 1.4]	0.9 ± 0.2 [0.7, 1.2]	1.5 ± 0.7 [0.8, 2.5]	1.0 ± 0.3 [0.6, 1.4]	1.0 ± 0.3 [0.5, 1.2]
DOR	1.7 ± 0.8 [0.8, 3.0]	1.0 ± 0.3 [0.6, 1.3]	1.3 ± 0.5 [0.6, 2.0]	0.6 ± 0.5 [0.0, 1.4]	1.4 ± 0.9 [0.6, 2.9]	1.5 ± 1.6 [0.5, 4.3]
Significant models	1	0	0	0	1	1
**LPO CV scheme**						
SE (%)	47.8 ± 6.1 [43.5, 56.5]	67.0 ± 7.9 [56.5, 73.9]	49.6 ± 8.5 [39.1, 60.9]	39.1 ± 9.2 [26.1, 47.8]	53.9 ± 6.6 [43.5, 60.9]	52.2 ± 6.9 [43.5, 60.9]
SP (%)	54.8 ± 6.6 [47.8, 60.9]	44.3 ± 10.8 [34.8, 60.9]	52.2 ± 5.3 [43.5, 56.5]	50.4 ± 12.5 [34.8, 69.6]	54.8 ± 5.0 [47.8, 60.9]	54.8 ± 12.9 [39.1, 69.6]
BAC (%)	51.3 ± 4.8 [45.7, 58.7]	55.7 ± 7.1 [45.7, 65.2]	50.9 ± 5.2 [43.5, 56.5]	44.8 ± 6.6 [37.0, 54.3]	54.3 ± 4.3 [50.0, 60.9]	53.5 ± 7.5 [43.5, 60.9]
PLR	1.1 ± 0.2 [0.8, 1.4]	1.3 ± 0.3 [0.9, 1.8]	1.0 ± 0.2 [0.8, 1.3]	0.8 ± 0.3 [0.5, 1.3]	1.2 ± 0.2 [1.0, 1.6]	1.2 ± 0.3 [0.8, 1.6]
NLR	1.0 ± 0.2 [0.7, 1.2]	0.8 ± 0.3 [0.5, 1.3]	1.0 ± 0.2 [0.8, 1.3]	1.3 ± 0.3 [0.9, 1.5]	0.8 ± 0.1 [0.6, 1.0]	0.9 ± 0.3 [0.6, 1.3]
DOR	1.2 ± 0.5 [0.7, 2]	1.9 ± 1.1 [0.7, 3.6]	1.1 ± 0.4 [0.6, 1.7]	0.7 ± 0.4 [0.3, 1.5]	1.5 ± 0.6 [1.0, 2.4]	1.5 ± 0.8 [0.6, 2.4]
Significant models	0	0	0	0	1	0
**5-fold CV scheme**						
SE (%)	42.6 ± 3.6 [39.1, 47.8]	40.9 ± 5.8 [34.8, 47.8]	59.1 ± 6.6 [52.2, 69.6]	45.2 ± 8.5 [34.8, 56.5]	53.0 ± 10.4 [39.1, 65.2]	57.4 ± 8.4 [47.8, 69.6]
SP (%)	54.8 ± 12.5 [34.8, 65.2]	40.9 ± 7.3 [30.4, 47.8]	40.9 ± 8.5 [30.4, 52.2]	53 ± 11.3 [34.8, 65.2]	54.8 ± 6.6 [47.8, 60.9]	53 ± 11.7 [39.1, 65.2]
BAC (%)	48.7 ± 6.6 [39.1, 56.5]	40.9 ± 2.4 [37.0, 43.5]	50.0 ± 5.1 [45.7, 56.5]	49.1 ± 2.5 [45.7, 52.2]	53.9 ± 5.2 [47.8, 60.9]	55.2 ± 9.3 [47.8, 67.4]
PLR	1.0 ± 0.3 [0.7, 1.4]	0.7 ± 0.1 [0.6, 0.8]	1.0 ± 0.2 [0.9, 1.2]	1.0 ± 0.1 [0.9, 1.1]	1.2 ± 0.2 [0.9, 1.6]	1.3 ± 0.5 [0.9, 2.0]
NLR	1.1 ± 0.3 [0.8, 1.6]	1.5 ± 0.2 [1.3, 1.9]	1.0 ± 0.3 [0.7, 1.3]	1.1 ± 0.1 [0.9, 1.3]	0.9 ± 0.2 [0.6, 1.1]	0.9 ± 0.3 [0.5, 1.1]
DOR	1.0 ± 0.5 [0.4, 1.7]	0.5 ± 0.1 [0.3, 0.6]	1.1 ± 0.5 [0.7, 1.8]	0.9 ± 0.2 [0.7, 1.2]	1.5 ± 0.6 [0.8, 2.4]	2.0 ± 1.6 [0.8, 4.3]
Significant models	0	0	0	0	0	1

Measures for each tested combination of brain regional feature type [i.e., regional-based gray (ROIGM) and white (ROIWM) matter volume; and surface-based regional cortical thickness, gyrification, sulci, and complexity indexes (ROISurface)], feature selection [i.e., no feature selection (No-FS); and forward feature selection (FFS)], and cross-validation (CV) scheme [i.e., leave-one scan acquisition protocol-out (LSO) CV; leave-one per group-out (LPO) CV; and 5-fold CV] are presented. Statistical significance of the balanced accuracy (BAC) for each bootstrapped sample was tested using permutation testing with a significance level of 5%. Data format: mean ± standard deviation [min max]. DOR, diagnostic odds ratio; NLR, negative likelihood ratio; PLR, positive likelihood ratio; SE, sensitivity; SP, specificity. Significant models: number of models with statistically significant BAC higher than 50%.

**TABLE 5:** Performance measures of each structural magnetic resonance imaging (SMRI) classification model based on voxel-wise features across bootstrapped samples.

	LSO CV scheme		LPO CV scheme		5-fold CV scheme	
	VBGM	VBWM	VBGM	VBWM	VBGM	VBWM
SE (%)	20.9 ± 34.8 [0, 82.6]	46.1 ± 38.2 [4.3, 78.3]	47.0 ± 10.4 [34.8, 60.9]	50.4 ± 11.3 [34.8, 60.9]	30.4 ± 10.2 [21.7, 43.5]	41.7 ± 8.5 [34.8, 56.5]
SP (%)	72.2 ± 35.7 [8.7, 91.3]	53 ± 35.4 [21.7, 95.7]	55.7 ± 8.4 [43.5, 65.2]	53.0 ± 7.1 [47.8, 65.2]	51.3 ± 7.8 [43.5, 60.9]	52.2 ± 6.1 [43.5, 60.9]
BAC (%)	46.5 ± 3.6 [43.5, 52.2]	49.6 ± 2.4 [45.7, 52.2]	51.3 ± 7.5 [45.7, 63.0]	51.7 ± 2.8 [47.8, 54.3]	40.9 ± 2.4 [37.0, 43.5]	47.0 ± 6.8 [41.3, 58.7]
PLR	0.6 ± 0.6 [0.0, 1.5]	0.9 ± 0.3 [0.3, 1.1]	1.1 ± 0.4 [0.8, 1.8]	1.1 ± 0.1 [0.9, 1.2]	0.6 ± 0.1 [0.5, 0.8]	0.9 ± 0.3 [0.7, 1.4]
NLR	1.3 ± 0.4 [1.0, 2.0]	1.0 ± 0.1 [0.9, 1.1]	1.0 ± 0.3 [0.6, 1.2]	0.9 ± 0.1 [0.8, 1.1]	1.4 ± 0.1 [1.3, 1.5]	1.1 ± 0.3 [0.7, 1.4]
DOR	0.7 ± 0.9 [0.0, 2.2]	0.8 ± 0.4 [0.1, 1.1]	1.3 ± 1.0 [0.7, 3.1]	1.1 ± 0.2 [0.8, 1.4]	0.4 ± 0.1 [0.2, 0.6]	0.9 ± 0.7 [0.5, 2.1]
Significant models	0	1	0	0	0	0

Measures for each tested combination of voxel-wise feature type [i.e., voxel-based gray (VBGM) and white (VBWM) matter volume maps], feature dimensionality reduction through principal component analysis and cross-validation (CV) scheme [i.e., leave-one scan acquisition protocol-out (LSO) CV; leave-one per group-out (LPO) CV; and 5-fold CV] are presented. Statistical significance of the balanced accuracy (BAC) for each bootstrapped sample was tested using permutation testing with a significance level of 5%. Data format: mean ± standard deviation [min max]. DOR, diagnostic odds ratio; NLR, negative likelihood ratio; PLR, positive likelihood ratio; SE, sensitivity; SP, specificity. Significant models: number of models with statistically significant BAC higher than 50%.

**TABLE 6:** Performance measures of: (1) a genetic schizophrenia polygenic risk score (PRS), (2) a list of psychosis-associated single nucleotide polymorphisms (SNPs), (3) expression quantitative trait loci (eQTL) scores **(43)** of a list of psychosis-associated genes expressed in the brain; (4) an environmental schizophrenia risk score (ERS), and (5) a list of schizophrenia-associated environmental risk factors, classification models across bootstrapped samples.

	PRS	SNP	eQTL score	ERS	Environmental risk factors
SE (%)	42.1 ± 20.0 [21.1, 63.2]	41.9 ± 13.6 [23.8, 61.9]	61.0 ± 17.0 [47.6, 85.7]	44.9 ± 5.1 [29.7, 56.8]	10.6 ± 4.9 [5.9, 17.6]
SP (%)	46.3 ± 11.4 [31.6, 57.9]	50.5 ± 16.4 [28.6, 66.7]	31.4 ± 23.2 [4.8, 57.1]	50.8 ± 8.2 [45.9, 64.9]	70.6 ± 7.2 [64.7, 82.4]
BAC (%)	44.2 ± 15.3 [26.3, 60.5]	46.2 ± 10.7 [33.3, 61.9]	46.2 ± 4.9 [40.5, 52.4]	47.8 ± 8.8 [37.8, 60.8]	40.6 ± 2.5 [38.2, 44.1]
PLR	0.9 ± 0.5 [0.3, 1.5]	0.9 ± 0.4 [0.5, 1.6]	0.9 ± 0.1 [0.8, 1.1]	1.0 ± 0.4 [0.6, 1.6]	0.4 ± 0.1 [0.2, 0.5]
NLR	1.4 ± 0.8 [63.6, 2.5]	1.3 ± 0.6 [0.6, 2.2]	1.9 ± 1.1 [0.9, 3.0]	1.1 ± 0.3 [0.7, 1.5]	1.3 ± 0.1 [1.1, 1.4]
DOR	1.0 ± 1.0 [0.1, 2.4]	1.0 ± 1.0 [0.2, 2.6]	0.7 ± 0.4 [0.3, 1.2]	1.0 ± 0.8 [0.4, 2.4]	0.3 ± 0.1 [0.2, 0.4]
Significant models	0	0	1	0	0

Statistical significance of the balanced accuracy (BAC) for each bootstrapped sample was tested using permutation testing with a significance level of 5%. Data format: mean ± standard deviation [min max]. DOR, diagnostic odds ratio; NLR, negative likelihood ratio; PLR, positive likelihood ratio; SE, sensitivity; SP, specificity. Significant models: number of models with statistically significant BAC higher than 50%.

## 4. Discussion

This study aimed to predict transition to psychosis from an ARMS using ML applied to quantitative data across modalities–i.e., neuroimaging (sMRI), genetics (genome-wide genotypes), and environment–collected when subjects first sought clinical help (i.e., at baseline) and were identified with an ARMS. This is, to the best of our knowledge, the first study: (1) of longitudinal design exploring sMRI, genetic and environmental data to predict the development of a psychotic disorder from a prodromal stage; and (2) when considering each modality individually, exploring a range of approaches (for genetics and environmental data) and/or feature combinations (for sMRI data).

### 4.1. Prediction of transition to psychosis using structural neuroimaging data

In this study we applied ML to structural neuroimaging data using a relatively larger sample and an ML approach, improved to the best of our ability, to detect transition to psychosis from an ARMS and to replicate previous positive findings of accuracies 74 to 84% of six studies, which together used 3 independent samples (10–15). For this, we decided: to: (1) use only the most recent versions of the image processing tools (i.e., CAT12) and ML tools (i.e., NeuroMiner); (2) replicate as accurately as possible the methods that were described in the abovementioned MRI papers since it was not possible to access their processing and ML pipelines; (3) add a layer of ML generalizability by bootstrapping and fitting a model to each subsample; and (4) overcome previous studies’ limitations (e.g., sample unbalancing for demographics). Furthermore, we explored, for the first time, the use of whole brain white matter volume and regional white matter volume, cortical thickness, and surface-based brain gyrification, sulci depth, and complexity indexes with ML to predict transition to psychosis.

Unexpectedly, we did not replicate previous findings. After balancing the samples for binary classification of transition to psychosis accounting for age, sex, and the three different scan acquisition protocols to avoid overoptimistic results, the performance of all tested combinations (i.e., of feature type–ROIGM, ROIWM, ROISurface, VBGM, or VBWM; feature manipulation–feature dimensionality reduction through PCA, no feature selection, or forward feature selection; and CV scheme–LSO CV, LPO CV, or 5-fold CV) were not significantly better than chance level.

Compared to the previous studies reporting high balanced accuracies (74 to 84%) in predicting transition to psychosis from sMRI maps (10–15), the current study has some advantages. First, this study’s sample is drawn from a more naturalistic ARMS population as it includes subjects whose sMRI images were acquired using three different scan acquisition protocols. Training a classification model with data from different centers potentially increases its generalizability. Only one of the previous transition to psychosis prediction studies used a two-site group balanced sample (12), combining the samples reported in two previous studies by the same authors (10, 11). The main differences between this report and our study are the following: (a) Their sample was larger than our balanced bootstrapped samples (i.e., 36% larger than ours, measured as the absolute value of the change in sample size, divided by the average of the size of the two samples). However, we tested our ML models on five balanced subsamples (i.e., through bootstrapping), allowing us to obtain a measure of generalizability of these models’ performance. Moreover, they do not present a measure of the statistical significance of the model’s BAC, which we do herein. (b) They controlled the effect of site on the classification using partial correlations during the training phase of the CV cycle, whereas we controlled it by keeping the same proportion of subjects at an ARMS that transitioned to psychosis and those who did not in each scan protocol during the training phase of the CV cycle (i.e., when using the LPO CV scheme as the previous study did). Additionally, we also guaranteed that the pair of subjects left out for testing/validation were from the same site. This potentially increases the generalizability of the classification model by training it with a more heterogeneous sample (and, as explained above, more naturalistic) and diminishing the effect of site on the testing/validation classification accuracy, which is not taken into account in the previous report (12).

Second, we trained our classification models with samples balanced for group (subjects at an ARMS who later transitioned to psychosis and who did not), age at scan and sex. Balancing for group is important to avoid biasing the classification model to the most represented group and it was not taken into account by three out of six previous reports (10, 11, 14). Moreover, the effects of age (49) and sex (50) on brain structure, rate of transition to psychosis from ARMS (2), and prevalence of psychosis (3, 51), have been consistently reported and, therefore, should be taken into account in these studies. All previous reports (and the current study) matched transition proportion for age and sex (10–14), except for one (15). Das and colleagues reported a statistically significant and better than chance level BAC in predicting transition to psychosis using a sample unbalanced for both group and sex. Although they used a ML algorithm with class (i.e., group) weighing–which in summary increases the influence of the minority class when training the model by assigning higher weights to rare cases, the authors performed an unspecified correction for sex effect (as well as for age and TIV effects) to the data during the training CV cycle. This approach may not be the most appropriate given the known effect of sex on brain structure (50) and the, abovementioned, empirically tested association between sex and group (i.e., transition to psychosis from an ARMS vs. no transition) (15), which makes sex a potential confounder in this analysis. Furthermore, in three of the six previous reports, the effects of age and sex were corrected before entering the ML analysis (10), and during the training CV cycle (11, 15) using partial correlations (10, 11) or an unspecified method (15)–which we did not perform. Correction for age effects in ML analysis has been previously shown to increase classification accuracy in Alzheimer’s disease, when it is estimated from healthy subjects (52). Correction for effects of no interest in ML analyses should be done with extreme caution as it can easily remove relevant subject-specific information (53). This is especially important when the correction is being performed in a non-healthy (i.e., non-standard) population, because the effect of external variables such as age and sex might be modulated by the presence of the disease (e.g., being at ARMS or having schizophrenia).

Third, this study’s sample is composed of subjects whose clinical diagnosis of an ARMS was based on having a schizotypal personality disorder or on the subject’s familial-high risk coupled with functioning decline and on the CAARMS (54), which mainly evaluates positive symptoms. These were not the same criteria as those used in the previous studies predicting transition to psychosis from an ARMS. These previous studies all used samples of subjects clinically assessed with tools that evaluate not only positive symptoms, but also basic and negative symptoms (10–12, 14, 15), except one (13), which included only familial-high risk subjects in its sample. This potentially increases the inclusion of subjects in the early phase of the psychosis prodrome (characterized by the presence of basic and negative symptoms), whereas our sample includes mainly subjects in the late prodromal phase of psychosis (characterized mainly by the presence of positive symptoms) (2). Therefore, our results suggest that previously reported accuracies in predicting transition to psychosis may be population-specific, poorly generalizable to differently clinically characterized populations (as ours herein).

### 4.2. Prediction of transition to psychosis using genetic data

In this study we applied ML to genetic data and used three types of genetic features to detect transition to psychosis from an ARMS: (a) a schizophrenia PRS that we have previously shown to distinguish FEP patients from healthy controls (26) and ARMS-T from ARMS-NT (16), (b) a set of psychosis-associated SNPs previously associated with schizophrenia in a recent GWAS meta-analysis (27), and (c) a brain-specific expression Quantitative Trait Loci (eQTL) score including the latter genes.

Genetic data showed a poor performance in predicting transition to psychosis from an ARMS. SNPs-based classification models have been previously shown to classify schizophrenia (18, 19, 21), and FEP patients (23) (vs. healthy controls) better than chance level, but not subjects at an ARMS vs. healthy controls or FEP patients (23). Furthermore, one of these studies has selected a list of SNPs from the Psychiatric Genomics Consortium 2 (PGC2) (21, 42), which potentially overlaps with the ones selected in this study (27).

Despite the (scarce) evidence of the potential of PRS for schizophrenia (20–22) to classify schizophrenia patients (vs. healthy controls) and the one report showing the schizophrenia PRS’s ability to predict transition to psychosis (16) we were not able to predict transition to psychosis from an ARMS using this type of genetic feature. Although the latter study (16) used a larger sample (i.e., 106% higher than ours, measured as the absolute value of the change in sample size, divided by the average of the size of the two samples) to train the PRS-based model, sample balancing in terms of group and age or sex were not taken into account or that was unclear, respectively. Furthermore, herein we applied a bootstrapped sample approach to estimate generalizability of the PRS-based model by assuring that each bootstrapped sample met the balancing conditions for group, age, and sex–which does not seem to be the case in that study (16). Furthermore, another possible explanation for the PRS negative results is that although the genetic architecture, conveyed through a PRS, has been shown to differ between patients with schizophrenia and healthy controls, one cannot exclude the possibility that it is specific to schizophrenia (a fully developed psychotic disorder), and might even be present in all subjects at an ARMS, i.e., those who later transition to psychosis and those who do not. The constellation of genetic variations (i.e., SNPs) that might confer susceptibility to transition to psychosis already from a prodromal stage is not necessarily the same as the one for schizophrenia (when drawn in comparison to healthy controls). This may justify the advantage of using a less hypothesis-based approach for the selection of genetic features (as we did by pre-selecting a large list of SNPs and performing an embedded feature selection using elastic net regression). Lastly, using a PRS formula made specifically for transition to psychosis from an ARMS would require a larger and independent sample to estimate SNP effect sizes, which might be better provided by multicenter projects, such as NAPLS 2 (55) and PRONIA^4^ over the next years.

Expression Quantitative Trait Loci (eQTL) scores for psychosis associated genes expressed in the brain were also not able to predict transition to psychosis from an ARMS. Only one previous study has shown the predictive value of gene expression profiling in the frontal brain region in classifying schizophrenia patients (vs. healthy controls) (17). In the present study, instead of actual gene expression measures we used a proxy for a-genetically regulated component of the expression of genes, the eQTL scores. Although we have computed eQTL scores only for the genes having a validated eQTL score model (43), this does not guarantee that the estimated gene expression represents (or correlates perfectly with) the real levels of the expression. Furthermore, although we have selected the initial list of genes as the ones most associated with schizophrenia (vs. healthy controls), this selection did not take into account the expression profile of these genes in the brain, and we have computed an eQTL score for several brain tissues. A future improvement of this step would be to test an eQTL scores-based model with a selection of genes that: (a) are highly expressed in the brain in healthy subjects, and (b) their expression is associated to a schizophrenia diagnosis, or even better with the transition to psychosis from an ARMS.

### 4.3. Prediction of transition to psychosis using environmental data

In this study we applied, for the first time, ML to environmental data using two types of features to detect transition to psychosis from an ARMS: (a) a schizophrenia ERS which we have previously reported (28), and (b) a set of environmental risk factors as predictors. Overall, neither environmental risk assessment, could predict transition to psychosis from an ARMS with an averaged accuracy, i.e., across bootstrapped samples, better than chance level. Although we know of no similar longitudinal ARMS transition study, the closest other report using ML and environmental data to diagnose schizophrenia (vs. healthy controls) (22) also found a BAC not statistically better than chance level, even having included features such as the presence of obstetric complications and of developmental anomalies, the parental socio-economic status; and –without feature selection–trained and tested the model in a 13 times larger, albeit age, sex, and group -unbalanced, sample (103 patients and 337 controls) than ours (22). However, due to the still poorly understood environmental risk mechanisms one cannot exclude the lack of statistical power as a potential explanation for these negative findings including ours.

The ML model trained with the ERS for schizophrenia, which we have tested as an (admittedly limited) exploratory predictor of the transition to psychosis from an ARMS, showed a poor performance, i.e., a BAC similar to chance level. Indeed, ERS is a composite score of individual risk factors computed under the assumption that the risk factors are completely independent (28), which has been shown not to be the case (56)–i.e., intercorrelated risk factors may inflate the ERS estimation. This crude approach may limit the ability of the ERS to capture the detailed environmental architecture underlying psychosis. Moreover, just as for a PRS, an ERS for schizophrenia may not be a good substitute of a potential ERS for transition to psychosis from an ARMS (57).

Lastly, our criterion for training and testing a fully multimodal ML model with modalities that would show an ML model performance statistically better than chance (i.e., 50%) predicting transition to psychosis from an ARMS in at least 3 of 5 bootstrapped samples was not fulfilled given that none of the modality-based ML models survived that threshold. This conservative criterion was chosen given the already small sample size available for the training of the multimodal ML model, i.e., only 6 ARMS-T and 23 ARMS-NT (only this subset of subjects had data for the three data modalities, simultaneously). The decrease in sample size, remarkably impairs the prediction power of the model, i.e., its accuracy. Without previous evidence of the ability to predict transition to psychosis from an ARMS by modality supporting its integration in a multimodal ML model, negative results from this multimodal model would be highly difficult to explain, as they could theoretically be explained by the increase of noise in the model due to the inclusion of features that did show previous predictive ability or by the lack of predictive power due to the very small sample size. Moreover, the parallel-to-ours, multi-site study, albeit very group-unbalanced (only 26 ARMS-T patients vs. 308 ARMS-NT), from the PRONIA project, showed that a stacked model combining similar data to our study’s plus human prognostic ratings could predict transition to psychosis with a balanced accuracy of 86% and a good geographical generalizability (25). This multimodal approach was showed to improve biological-based unimodal models by 15% (VBGM volume maps-based model) and 20% (PRS for schizophrenia-based model). As such, the replication of this promising finding, following the same multimodal approach as that study, using in our study’s sample and data features co-existing in both samples, would be interesting as an additional method to ascertain whether our negative findings are due to lack of power or to no discriminability with our feature sets.

### 4.4. Limitations

This study was limited by several factors. First, and foremost, the small sample size may have limited the performance of classification models, even though our sample size was informed by previous ML studies showing 74–84% accuracies in predicting transition to psychosis from an ARMS (10–15). Indeed, this is a critical limitation when dealing with high dimensional data, such as neuroimaging and genetics–which we have used herein. Although we have taken measures to avoid overfitting and an overestimation of the classification models’ performance such as artificially increasing the sampling through bootstrapping and employing CV strategies, this might not be enough to overcome this limitation. Indeed, our complementary analysis comparing the models’ training and testing performance (results in the Supplementary material) is indicative that some of the tested classification models (mainly trained with neuroimaging or with SNPs) might suffer from some degree of overfitting. Ultimately, we cannot determine whether our negative findings were due to lack of power to obtain a good performance or due to a true lack of association between the predictors and the transition to psychosis from an ARMS (and hence inflated findings from previous studies). This is one of the reasons why replication studies in independent datasets are essential in ML literature. As a final note, a power analysis for this study design would have been the most informative way to define the sample size needed to achieve an accuracy in predicting transition to psychosis from an ARMS better than chance level. However, this is not a trivial task in ML analysis and there is no established method to perform this analysis as there is for univariate analysis [for examples of studies exploring innovative ways of computing sample size for classification problems see Refs. (58, 59)] and, therefore, it was not performed.

Second, in order to dilute possible confounding effects in the developed classification models we have restricted the samples used to train the models to: (a) be class-balanced, i.e., with the same number of ARMS-T and ARMS-NT subjects; (b) be matched for age, sex, scanning acquisition protocols for neuroimaging data; (c) include subjects with European ancestry only for genetic data; and (d) limit the proportion of missing data for the environment data. Although this has artificially homogenized the study sample thus avoiding the presence of statistical confounders, it has deemed the sample to be less representative of the ARMS population. Third, overall, the findings of this study are only valid to young help-seeking individuals, i.e., that are clinically screened for ARMS criteria, and whose ARMS diagnosis was based on having a schizotypal personality disorder or on the subject’s familial-high risk coupled with functioning decline and on the CAARMS (54), which mainly evaluates positive symptoms.

## 5. Conclusion and future directions

In this study, we explored the value of using exclusively quantitative and multimodal data (i.e., as predictors) to predict transition to psychosis from an ARMS. Overall, we found that, contrary to what has been previously reported, sMRI could not predict transition to psychosis from an ARMS. We have employed several ML strategies aiming to replicate the highly promising previous positive sMRI findings (74–84%) (10–15). This is even though our sample was larger than four of the above 6 studies (10, 11, 13, 14), respectively (Conversely, our sample was smaller than two of the above studies [Das et al. (15); Koutsouleris et al. (12), respectively]. This points to the need for a cautious interpretation of small sample size studies. Also, we could not replicate the one previous evidence of the value of the schizophrenia PRS in predicting transition to psychosis. Moreover, and to the best of our knowledge, we explored for the first time the value of environment in the prediction of psychosis already from a prodromal stage. Lastly, the genetic and the environmental data used could not predict transition to psychosis from an ARMS. In summary, the present study should serve as a call for caution and skepticism regarding the currently achievable prognostic and diagnostic biomarker development goals, with the existing modeling tools and data measurement tools. Additionally, our study’s methodological approaches tailored to each data modality, may serve as suggestive proofs-of-concept for the exploration of future multimodal datasets, either for novel discovery or replication of previous promising findings, across psychiatric disorders, not exclusive to ARMS. We further suggest larger samples (in the several hundreds) should be employed for both model training and testing, given the inherent high data dimensionality (specially of neuroimaging and genetics) and the still little established relevance of individual features. Although heterogeneity in phenotypic measurements is increased in larger samples, they bring not only statistical power but ecological generalizability, and thus carry a higher potential to be clinically useful. This is best achieved with consortia multi-center studies which are increasingly common albeit not without challenges (60). Alternatively, methods for synthetic generation of data such as the Generative Adversarial Networks (GAN)-based are also a promising avenue for sample size augmentation, now starting to be applied in the clinical research field (61). Last, but not least, we recommend the use of objective and quantitative criteria-based tools for the assessment of a ML biomarker’s clinical applicability, once high effect size and accuracy estimates are achieved, such as one we have previously proposed (62).

## Data availability statement

The datasets presented in this article are not readily available because ethics approval did not include public data sharing. Requests to access the datasets should be directed to the corresponding author.

## Ethics statement

The studies involving human participants were reviewed and approved by NHS South East London Research Ethics Committee. The patients/participants provided their written informed consent to participate in this study.

## Author contributions

VT ran most data preprocessing and statistical analyses and drafted the manuscript. EV coordinated genotyping and advised the genetic and environmental data analysis. AM and HF provided advise on imaging data processing and machine learning analysis. JS and IV collected imaging data. GB provided advise on imaging data processing. DP collected genetic and environmental data, co-designed the study, ran preliminary data preprocessing and machine learning analyses, and supervised the study. All authors revised the manuscript and agreed with its final version.

## Footnotes

1
http://www.neuro.uni-jena.de/cat/

2
http://www.fil.ion.ucl.ac.uk/spm/

3
http://proniapredictors.eu/neurominer/index.html

4
http://pronia.eu
