# Supplementary material for: Prediction of transition to psychosis from an at-risk mental state using structural neuroimaging, genetic, and environmental data
Source: Front Psychiatry. 2023 Jan 19;13:1086038. doi: 10.3389/fpsyt.2022.1086038 (PMC9892839; doi:10.3389/fpsyt.2022.1086038)
Supplement: Supplementary file 1 [file Data_Sheet_1.docx]

**Prediction of transition to psychosis from an ARMS using structural neuroimaging, genetic and environmental data**

**Supplementary material**

# **Supplementary methods**

## **Sample description**

The total sample consisted of 246 individuals at an ARMS, recruited at first presentation from consecutive referrals to the Outreach and Support in South London (OASIS) high-risk service, South London and Maudsley NHS Foundation Trust (1). OASIS is a clinical service located in Lambeth, South London, that offers treatment to individuals between 14 and 35 years of age who meet the ARMS criteria for psychosis. The presence of the ARMS was assessed using the comprehensive assessment of the ARMS (CAARMS), a detailed clinical assessment (2). In particular, an individual can meet the criteria for the ARMS if 1) had a recent decline in function coupled with either schizotypal personality disorder or a first degree relative with psychosis, examined using the family interview for genetic studies (FIGS) (3); or 2) experienced attenuated positive psychotic symptoms; or 3) had a Brief Limited Intermittent Psychosis (BLIP), a brief psychotic episode of less than 1 week’s duration that resolved without antipsychotic medication. Exclusion criteria applied to all participants was a history of significant head injury and current (in the last 12 months) substance dependency according to DSM-IV diagnostic criteria.

When the subjects were first diagnosed as being at an ARMS (i.e., baseline) a set of data were acquired: a) a structural Magnetic Resonance Imaging (sMRI s)can; b) genome-wide genotypes; and c) environmental risk factors assessments. Subjects were labeled as transitioned to psychosis (ARMS-T) if they later presented a first episode of psychosis (FEP) or as not-transitioned to psychosis (ARMS-NT) if they did not present a FEP within at least a period of 2 years. Furthermore, transition to psychosis during the follow-up period was established according to the Diagnostic and Statistical Manual of Mental Disorders, Fourth Edition (DSM-IV) (4) criteria based on clinical consensus between at least two experienced psychiatrists. Additional socio-demographic and clinical measures were also assessed at baseline, including age, sex, handedness, self-reported ethnicity, full scale intelligence quotient measured by National Adult Reading Test (5), years of education, and global assessment of function using Global Assessment of Functioning tool (GAF; at baseline and at follow-up) (4), and CAARMS (at baseline and follow-up) (2).

## **Structural neuroimaging data**

### Structural magnetic resonance imaging subsample

Two ARMS-T were scanned 68 and 136 days after the transition to psychosis date. Most of the ARMS group (84/99; 85%) were naïve to antipsychotics at the time of scanning; the remaining 15 (15%) had been exposed to antipsychotics.

### Structural magnetic resonance imaging acquisition

sMRI scans were acquired with two different scanners (one with a 1.5T strength and another with 3T) using three enhanced fast gradient echo 3-Dimensional (efgre3D) protocols. For a detail: a) acquisition protocol 1 – 1.5T Signa scanner (General Electric Medical Systems, USA; voxel size = 0.9 x 0.9 x 1.5 mm^3^; matrix of acquisition = 256 x 256 x 124; field-of-view = 220 mm; gap = 0 mm; repetition/echo/inversion times = 15.9 s/5.2 s/300 s; flip angle = 20°; 19 scans: 3 ARMS-T, 16 ARMS-NT); b) acquisition protocol 2 – 1.5T Signa scanner (General Electric Medical Systems, USA; voxel size = 0.9 x 0.9 x 1.5 mm^3^; matrix of acquisition = 256 x 256 x 124; field-of-view = 220 mm; gap = 0 mm; repetition/echo/inversion times = 21.3 s/5.1 s/0 s; flip angle = 20°; 33 scans: 14 ARMS-T, 19 ARMS-NT); and c) acquisition protocol 3 – 3T Signa scanner (General Electric Medical Systems, USA; voxel size = 1.1 x 1.1 x 1.1 mm^3^; matrix of acquisition = 256 x 256 x 146~196; field-of-view = 280 mm; gap = 1.1 mm; repetition/echo/inversion times = 7.0~9.6 s/2.8~2.9 s/450 s; flip angle = 20°; 33 scans: 14 ARMS-T, 19 ARMS-NT).

### Image processing

T1w images were processed with Computational Anatomy Toolbox (CAT12; v1092, <http://www.neuro.uni-jena.de/cat/>), a Statistical Parametric Mapping 12 (SPM12) add-on (v6909, <http://www.fil.ion.ucl.ac.uk/spm/>) using default settings and MATLAB (9.3) using the same pipeline as described in (6). In summary, the preprocessing included bias field inhomogeneity correction; image segmentation into grey matter, white matter, and cerebrospinal fluid; spatial normalization to a template derived from 555 healthy subjects of the IXI-database (<http://www.brain-development.org>) using the Diffeomorphic Anatomical Registration using Exponentiated Lie algebra (DARTEL) algorithm (7); and a spatial smoothing of Full Width at Half Maximum (FWHM) of 8 mm. Finally, grey and white matter volumes for 64 regions of interest (ROI; description of each ROI is in the **STable 1**) were computed by dividing the volume of grey and white matter of each ROI (i.e. the sum of all voxels classified as grey or white matter inside that ROI, respectively) by the total intracranial volume (TIV; the sum of all voxels classified as grey or white matter or as cerebrospinal fluid). ROIs were defined by the Hammers atlas (8).

Additionally, the segmented images (i.e. grey and white matter images) were used to estimate cortical thickness and central surface using the projection-based thickness method. Finally, regional-based cortical thickness and surface measures (i.e. folding measures) – gyrification index, i.e. the absolute mean curvature (9), the depth of sulci and the measurement of local surface complexity, i.e. the fractal dimension (10), were extracted for 68 ROIs (description of each ROI is in the **STable 2**) defined by the Desikan-Killiany atlas (11).

### Image quality control

The quality of each processed image (which is decomposed in several volume- and surface-based brain measures as described **Image processing**) was empirically assessed using the quality assurance framework of CAT12. This framework describes the properties of the image before CAT12 processing and estimates four qualitative measures using the tissue segmentation results: a) noise contrast ratio (NCR) – it yields information regarding the amount of noise in the image by measuring the local standard deviation in the optimized white matter segment scaled by the minimum tissue contrast; b) inhomogeneity contrast ratio (ICR) – it yields information regarding the bias field inhomogeneity measured as the global standard deviation within the optimized white matter segment scaled by the minimum tissue contrast; c) root-mean-squared resolution (RES) – it described the resolution of the image by measuring the root-mean-square of the voxel size; and d) weighted average image quality rating (IQR) – averaging the above three measures. The quality ascertainment framework maps the rating scores to image quality grades: from A (excellent) to F (unacceptable/failed). We set the subject’s image inclusion threshold at D (sufficient), i.e. only subjects which images had a IQR of A to D were included in the final sample, as it has been shown that typical scientific data (clinical) data get good to satisfactory ratings (12). NCR, ICR, RES and IQR were analyzed using Univariate analysis of variance (ANOVA) to test for the effects of ‘Protocol’, ‘Transition’ and ‘Protocol x Transition’ using SPSS.

## **Genetic data**

### Genotyping and imputation

Genotyping procedures have been previously described (13,14). In detail, DNA was extracted from blood or cheek swabs. The samples were genotyped either at the South London and Maudsley (SLaM) NHS Foundation Trust/King’s College London Biomedical Research Centre Genomics Laboratory on the Illumina HumanCore Exome BeadChip (“SLaM sample”, 935 subjects from which 134 were ARMS – 40 ARMS-T and 94 ARMS-NT) or at the Wellcome Trust Sanger Institute (WTSI, Cambridge, UK) on the Genome-wide Human single nucleotide polymorphism (SNP) Array 6.0 (“WTSI sample”, 786 subjects from which 1 was ARMS-T). Quality control (QC) included exclusion of SNPs with minor allele frequency (MAF) <1% or 2%, SNPs with genotypic failure >1% or >5%, and individuals with genotypic failure >1% or 2%, and SNPs with Hardy Weinberg equilibrium p<10^-5^ in healthy subjects only or p<10^-6^ in SLaM or WTSI sample, respectively. Imputation was performed with IMPUTE2 (15) based on the 1000 Genomes phase 3 reference panel (16). The imputed markers underwent a second stage of QC to exclude SNPs that were missing in >5% or 1% of individuals in SLaM or WTSI sample, respectively, or had imputation information score (INFO) <0.8.

### Genotyped samples merging and population stratification analysis

SLaM and WTSI samples were merged keeping only overlapped imputed SNPs. To account for genotyping and imputation QC differences in the two samples and following standard genome-wide association study (GWAS) QC guidelines (17), an extra QC was run excluding SNPs missing in >2% of individuals and with a MAF <5%. After the QC, a population stratification analysis was conducted to select only subjects with an European ancestry (the number of subjects per self-reported ethnicity is in the **STable 3**). In detail, a principal component analysis was applied to a) the merged total sample (i.e. 935 subjects from the SLaM sample and 786 subjects from the WTSI sample); and b) the 1000 Genomes phase 3 dataset (i.e. 2504 subjects). Then, the first two principal components from each dataset were extracted and plotted against each other (**SFigure 1**). The 1000 Genomes dataset is used in this analysis as a reference for the genetic structure across populations. It is comprised by five super populations: African, American, East Asian, European, and South Asian ancestry. As the genetic ancestry of the subjects in the reference dataset is known, the ancestry of the subjects from a non-reference dataset can be inferred by visually comparing the two first principal components plotted against each other with those from the reference dataset. For this study only subjects with an European ancestry were selected for further analysis according to the following criteria: a) subjects had to have reported as being ‘white’ (i.e. self-reported ethnicity is a proxy of the genetic ancestry); and b) subjects had to show a genetic structure explained by the first two components roughly in a similar manner as in the reference dataset, i.e. the scatter plot had to be visually similar. Seventy-five subjects met these criteria (21 ARMS-T and 54 ARMS-NT) and two subjects were excluded as they have reported as being white, but showed a genetic structure similar to other populations, i.e. Asian and African (**SFigure 1**). Samples merging, QC and population stratification analysis were conducted using PLINK 1.9 (<https://www.cog-genomics.org/plink2>) (18).

### Polygenic risk score extraction

The polygenic risk score (PRS) was computed for each subject (${PRS}_{i}, i=1,\ldots70$) in this study’s sample (i.e. ARMS sample) as the sum of the alleles of the SNPs that have been previously associated with the diagnosis of schizophrenia (versus healthy controls; ${SNP}_{j}, j=1,\ldots,m$), weighted by their effect size, $\ln\left( {OR}_{{SPN}_{j}}, j=1,\ldots,m \right)$, in that association (**SEq. 1**). The association between each SNP and the schizophrenia diagnosis was conducted by the Psychiatric Genomics Consortium GWAS meta-analysis study (19) in a sample of 36 989 patients with schizophrenia and 113 075 healthy controls. The number of SNPs to be include in the PRS was determined by Vassos and colleagues the ones below an association with schizophrenia *p*-value below 0.1. The reason this *p*-value threshold was chosen is that it explained the highest proportion of variance between the PRS and the diagnosis of FEP (*vs.* healthy controls) (20). The actual computation of the PRS for the ARMS sample was done by Dr. Evangelos Vassos at the Institute of Psychiatry, Psychology and Neuroscience, King’s College London, United Kingdom, and was only available for 70 ARMS subjects (19 ARMS-T and 51 ARMS-NT).

${PRS}_{i}=\sum_{j}^{m} \ln\left( {OR}_{{SPN}_{j}} \right){SNP}_{j}$ (**SEq. 1**)

### Psychosis-associated SNPs

The SNP sets to be included in the machine learning (ML) analyses were defined as follows. First, SNPs were ranked by their psychosis association significance (i.e. *p*-value) according to the (21) study. Then, four subsets were defined by selecting all the SNPs meeting a statistical significance level threshold (several thresholds were tested: *p* < 10^-2^, *p* < 10^-3^, *p* < 10^-4^, or *p* < 10^-5^). The number of SNPs from the original study and the ones available in our sample are in the **STable 4**. The maximum number of initial features, i.e. SNPs, to be included in the ML analyses is 79 247.

### eQTL scores of psychosis-associated genes

The expression quantitative trait loci (eQTL) score of a list of psychosis-associated genes identified by the same study from which the list of SNPs was extracted ((21); see also **2.5.2.2.1. Psychosis-associated SNPs**) was computed using the eGenScore tool (22). In specific, from a total of 571 genes that have been identified in the previous GWAS meta-analysis (21), 426 genes were available in the BrainEAC database (the training database of the eGenScore tool; (22,23); <http://www.braineac.org/>). Then, an eQTL score, which represents the predicted gene expression, for each gene and each brain tissue available in BrainEAC (i.e. frontal, temporal, and occipital cortices; putamen; substantia nigra; hippocampus; cerebellum; and white matter) was computed as follows. The sum of the alleles of the SNPs showed to be statistically associated with gene expression (in the brain) in the training database (BrainEAC) was weighted by their association effect size ($\beta$) adjusted to the expected proportion of those alleles in a standardized population, i.e. using the 1000 Genomes phase 3 dataset (16) (**SEqs. 2** and **3**). For each SNP (SNP_i_), this adjustment centers the expected contribution of the SNP_i_ at zero. In this way, replacing a missing genotype for a given SNP with zero sets the contribution of this SNP to its expected contribution in the population (24).

${eQTL score}_{j}=\sum_{i} \left( \left( \beta_{i}\times{SNP}_{i}-{ajustment factor}_{i} \right)\times{CalledSNP}_{i} \right)$ (**SEq. 2**)

where ${CalledSNP}_{i}=\left\{ \begin{aligned} 1, if the genotype of{SNP}_{i} is present \\ 0, if the genotype of {SNP}_{i} is missing \end{aligned} \right.$

$${adjustment factor}_{i}=\beta_{i}\times{Proportion}_{1 ref allele; i}+$$

${2\times\beta}_{i}\times{Proportion}_{2 ref allele;i}$ (**SEq. 3**)

Furthermore, only eQTL scores for which there was a model that have showed (1) a statistically significant internal validation performance (i.e. *p* < .05) and (2) an absolute Pearson correlation coefficient between observed gene expression and eQTL score of 0.1 at the internal validation were considered for the ML analysis (for details regarding eQTL score model significance see (22)). The number of genes per brain tissue for which an eQTL score was computed is in **STable 6**. The maximum number of initial features, i.e. number of genes for which an eQTL score was computed, to be included in the ML analyses is 141.

## **Environmental data**

Each subject was assessed on, at least one of, eight environmental risk factors: 1) tobacco and 2) cannabis consumption; 3) being migrant; 4) belonging to an ethnic minority; 5) the upbringing urbanicity level; 6) the parental age at birth; 7) the presence of childhood trauma; and 8) the season of birth. Exposure to tobacco was binarily defined as any exposure if the subject used to smoke at least one cigarette a day or as no exposure if otherwise. Exposure to cannabis was assessed in three main domains: consumption onset (late (score 1)/early (score 2) onset), frequency (infrequent (score 1)/frequent (score 2)) and duration (short- (score 1)/long (score 2)-term use). A composite score was computed as the sum of the consumption onset, frequency and duration scores (possible cannabis consumption score range: [0, 6]). Subjects belonged to an ethnic minority if they have self-reported has being as other than white or as white, but migrant. Therefore, belonging to an ethnic minority score was defined as 0 for native subjects, 1 for white migrants, 2 for self-reported black ethnicity and 3 for other self-reported ethnicity (e.g. asian, mixed). Risk for psychosis was defined as the lowest if the subject was upbrought in a village and highest if upbrought in a city, with being upbrought in a town conferring an intermediate risk (i.e. score 1 for village, 2 for town and 3 for city). Childhood trauma was assessed using the self-reported childhood experience of care and abuse questionnaire (25). Risk for psychosis due to exposure to childhood trauma was defined as a sum of exposure to the following events weighted by the number of years of exposure of each event: a) being bullied, b) being hit repeatedly, c) having seen or heard family violence, d) being separated from a parent for more than 1 year, and e) having been institutionalized. Finally, risk for psychosis was defined as higher if the subjects were born in the summer or autumn (score of 2) compared to being born in the winter or spring (score of 1).

### Environmental risk score extraction

The environmental risk score (ERS) was computed for each subject (${ERS}_{i}, i=1,\ldots134$) in this study’s sample as the sum of the exposure to each environmental risk factor that have been previously associated with the diagnosis of schizophrenia (*vs.* healthy controls; ${environmental risk factor}_{j}, j=1,\ldots,7$) weighted by their effect size, i.e. relative risk, ${RR}_{{environmental risk factor}_{j}}, j=1,\ldots,7$, in that association and adjusted by the average exposure to the risk factor in the general population, $\sum_{k}^{m} {RR}_{k}\times p_{k}$, (**SEq. 4**).

${ERS}_{i}=\sum_{j}^{7} \left( \log\left( \frac{{RR}_{{environmental risk factor}_{j}}}{\sum_{k}^{m} {RR}_{{environmental risk factor}_{j},k}\times p_{{environmental risk factor}_{j}k}} \right)\times10 \right)$ (**SEq. 4**)

In more detail, 7 environmental risk factors were included in the ERS (i.e. tobacco and cannabis consumption; belonging to an ethnic minority; the upbringing urbanicity level; the parental age at birth; the presence of childhood trauma; and the season of birth). For each factor, the most recent meta-analysis assessing the risk that the exposure to each factor confers to psychosis was selected and its effect size was extracted. Furthermore, whenever available, crude effect sizes or with minimum adjustment were selected and considered as relative risk (RR) even if presented as odds ratio, incidence rate ratios or RR. Moreover, these three effect size measures are good approximations of RR because psychosis is a rare outcome (i.e. psychosis as a diagnosis *vs.* healthy controls). The RRs in the meta-analyses are defined by comparing exposed with non-exposed individuals, with the risk factor defined as a binary or an ordinal variable. Then, the effect sizes are scaled to the averaged individual, i.e. by extracting the expected proportion of individuals in each level of the risk factor in the general population. This scaling is done under the assumption that only a minority of the population would not be exposed to any risk. The next step was to multiply the logarithm of the scaled RRs by 10 and to round them by the nearest integer. Finally, ERS was computed as the sum of each of the risk factor component, where missing values, i.e. missing risk factors, were replaced by zero. **STable 7** presents the information regarding each environmental risk factor, including the meta-analysis from which the exposure risk effect size was computed, the proportion of the general population that is exposed to the risk (by level of exposure), the exposure risk effect size scaled by this general population proportion, and the contribution of each risk factor (by level of exposure) to the ERS. The selection of the meta-analysis from which the effect sizes were extracted, definition of each factor’s exposure levels, and the general population exposure to the factor was originally presented in (24) for all the risk factors except for tobacco consumption and season of birth. For this last one, I followed the same methods as described in (24) to select the meta-analysis, define the level of exposure and extract the proportion of the general population that is exposed to the risk (by level of exposure). Only subjects with less than 20% of missing information (i.e. missing data for less than 2 environmental risk factors) were considered for the ML analysis. Therefore, the final sample included 37 ARMS-T subjects and 97 ARMs-NT subjects.

## **Feature dimensionality reduction with principal component analysis**

Feature dimensionality reduction was performed for VBGM and VBWM features using robust principal component analysis (PCA) (26,27). This algorithm projects the correlated voxels within the voxel-based maps to a given number of uncorrelated principal components, which represent compact sets of neuroanatomical features with reduced content of noise. Furthermore, these principal components are linear combinations of the correlated voxels computed to maximize the variance of the data. Robust PCA is specially designed to deal with non-symmetrical data, i.e., data under the influence of outliers, and is more suitable when the data is characterized by a much higher number of features than observations, such as us herein. Moreover, the main advantages of PCA are the reduction of a) the computational complexity of classification caused by the high dimensionality of structural MRI data; and b) the generalization error of classification by optimizing the number of principal components for data projections, thus maximizing the degree of anatomical information while minimizing the impact of noise. Here the robust PCA was applied during the inner cross-validation (CV) cycle (see **2.5.5. Cross-validation** in the main manuscript). The number of principal components that were retained explained up to 80% of the variance in the data and were limited by the inner CV cycle’s sample size, $n$, i.e., a maximum of only $n/2$ components could indeed be extracted. **STable 5** shows the maximum number of principal components that can be extracted for each inner CV cycle in each CV scheme that was used (see also **2.5.5. Cross-validation** in the main manuscript).

## **Elastic net for classification**

Binary classification of transition to psychosis from an ARMS (i.e. ARMS-T vs. ARMS-NT) using genetic (psychosis-associated SNPs or eQTL scores of psychosis-associated genes) or environmental (environmental risk factors) data was performed using logistic regularized regression with elastic net (28). This regularization method applies L1 ($\left\| \beta\right\|_{1}$; from least absolute shrinkage and selection operator (LASSO) regression) and L2 ($\left\| \beta\right\|^{2}$; from the ridge regression) penalties when estimating the weights of each predictor ($\beta$) in the regression (**SEq. 5**). L1 penalty can shrink coefficients to zero, which can help to reduce overfitting and perform feature selection. However, if a group of predictors are collinear, L1 penalty tends to select only one of the predictors, while ignoring the others (i.e. shrinking their coefficients to zero). Moreover, it can select at most the same number of predictors as the number of observations used to fit the regression model. These limitations are overcome by adding the L2 penalty, which also shrinks the coefficients, but in the case of collinearity it equalizes the coefficients of that group of predictors (i.e. instead of selecting only one). This helps to reduce the model complexity and multi-collinearity. The final elastic net penalty to be applied to the regression coefficients is defined by $l_{1}$ and $\lambda$. If $l_{1}=1$, then only L1 penalty would be applied, whereas if $l_{1}=0$, then only L2 penalty would be applied. Moreover, $\lambda$ is a numerical value that defined the amount of shrinkage to be applied to the regression coefficients. Herein, a search was carried out to identify the optimal $l_{1}$ and $\lambda$ values (i.e., $l_{1}=0:0.1:1; \lambda=0.01:0.01:1$) in the inner CV cycle (see **2.5.5.** **Cross-validation**). Furthermore, the implementation of the elastic net was done using the ‘glmnet’ v4.0 R package.

$\hat{\beta}={\arg min}_{\beta}\left\| y-X\beta\right\|^{2}+\lambda\left( l_{1}\left\| \beta\right\|_{1}+l_{2}\left\| \beta\right\|^{2} \right)$ (**SEq. 5**)

## **Logistic regression for classification**

Binary classification of transition to psychosis from an ARMS (i.e. ARMS-T vs. ARMS-NT) was performed using logistic regression. Logistic regression is a regression for binary outcomes (as herein). In the case where we have only one predictor, *X*, as I have herein, the probability of observing the outcome is *Y* is given by **SEq. 6**.

$P\left( Y \right)=\frac{1}{1+e^{-\left( \beta_{0}+\beta_{1}X \right)}}+\varepsilon$ (**SEq. 6**)

For binary classification, a threshold of 0.5 is commonly applied to the probability of observing the outcome. For example, a subject at an ARMS with a $P\left( Transition to psychosis \right)>0.5$ would be predicted to transition to psychosis, while a subject at an ARMS with a $P\left( Transition to psychosis \right)<0.5$ would be predicted to not develop a psychotic disorder, i.e. would not transition to psychosis.

## **Cross-validation**

**sMRI data.** Each classifier was trained in a nested-CV scheme for hyperparameter tunning (in the inner CV cycle) and to estimate the generalizability of the trained prediction model and its performance (in the outer CV cycle). The sample was split into *k* non-overlapping folds and each fold was iteratively used to estimate the performance of the classifier on validation data, while the remaining k-1 folds were used to train the decision rule of the classifier. Specifically, the purpose of the inner CV cycle is to use the training and testing subsamples to optimize a) feature manipulation, i.e., to find the optimal number of principal components to extract from the original feature set (i.e., feature dimension reduction with PCA when using VBGM or VBWM), or to select the optimal feature set (i.e., greedy forward feature selection when using ROIGM, ROIWM or ROISurface); and b) the linear SVM hyperparameter C. The outer CV cycle is used to estimate the generalizability of the trained prediction model by measuring the model performance on the validation subsample. Herein, we tested three different nested-CV schemes: a) leave-one scan acquisition protocol-out (LSO), b) leave-one per group from the same scan acquisition protocol-out (LPO), and b) classical 5-fold CV. Furthermore, the optimal hyperparameters/feature set were chosen as the ones yielding the highest balanced accuracy (see below) across the inner CV cycle.

**Genetic (PRS) and environmental (ERS) data.** The logistic regression was trained and tested in a simple leave-one per group-out (LPO) CV scheme.

**Genetic (psychosis-associated SNPs or eQTL scores of psychosis-associated genes) and environmental (environmental risk factors) data.** The elastic net logistic regression was trained using a nested-CV scheme. In summary, a 5-fold CV was applied to the inner cycle to find the optimal number of initial SNPs to be included in the model (i.e. defined by the statistical significance threshold *p* = 10^-2^, *p* = 10^-3^, *p* = 10^-4^, or *p* = 10^-5^, when using the psychosis-associated SNPs’ alleles as predictors) and the elastic net hyperparameters $l_{1}$ and $\lambda$. The optimal hyperparameters/feature set were chosen as the ones yielding the highest balanced accuracy (see below) across the inner CV cycle. The outer CV cycle is used to estimate the generalizability of the trained prediction model and was done with a LPO scheme.

## **Performance measures**

The classifier’s performance was evaluated using measures derived from the confusion matrix: sensitivity (SE), specificity (SP), balanced accuracy (BAC), positive likelihood ratio (PLR), negative likelihood ratio (NLR), and diagnostic odds ratio (DOR). SE (also known as the true positive rate) reflects the proportion of ARMS-T individuals (positive class) that have been correctly identified (**SEq. 7**), whereas SP (also known as the true negative rate) measures the proportion of ARMS-NT (negative class) that have been correctly identified (**SEq. 8**). BAC represents the average of individuals that are correctly identified as being ARMS-T or ARMS-NT (**SEq. 9**). PLR translates the probability of an ARMS-T being identified as an ARMS-T divided by the probability of an ARMS-NT being identified as an ARMS-T (**SEq. 10**), whereas NLR translates the probability of an ARMS-T being identified as an ARMS-NT divided by the probability of an AMRS-NT being identified as an ARMS-NT (**SEq. 11**). Finally, DOR is a measure of how efficient the diagnostic test (i.e. the classifier) is. It is measured by dividing the PLR by the NLR. All the performance measures were measured using the validation subsamples in the outer CV cycle.

$SE= \frac{true positives}{positives}$ (**SEq. 7**)

$SP= \frac{true negatives}{neagtives}$ (**SEq. 8**)

$BAC= \frac{SE+SP}{2}$ (**SEq. 9**)

$PLR= \frac{SE}{1-SP}$ (**SEq. 10**)

$NLR= \frac{1-SE}{SP}$ (**SEq. 11**)

## **Comparison between training and testing model performance**

The difference between the testing (i.e., from the inner CV cycle) and validation (i.e., from the outer CV cycle) (for the models trained with neuroimaging, genetic (SNPs or eQTL scores) or environmental (environmental risk factors) features) or between the training and testing (i.e. simple CV scheme) (for the models trained with genetics (PRS) or environmental (ERS) features) BAC was assessed with a paired two-sided Wilcoxon signed rank test and through Bland-Altman plots for each tested combination of feature type, feature manipulation, and CV strategy and across bootstrapped samples. The difference between the testing and validation BAC (or training and testing BAC in the case of a simple CV scheme) was considered statistically significant at a significance level of 5% (i.e., *p* < .05). Moreover, the effect size of the difference was extracted by computing the Cohen’s *d* for paired samples (**Eq. 7**), where $\bar{x}_{validation}$ and $\bar{x}_{testing}$ represent the mean validation and testing BAC, respectively; $s_{validation}$and $s_{testing}$ represent the standard deviation of the validation and testing BAC, respectively; and $r$ the Pearson correlation coefficient between the validation and testing BAC.

$\mathrm{Cohe}n^{'}s d= \frac{\bar{x}_{validation}-\bar{x}_{testing}}{\sqrt{s_{validation}^{2}+s_{testing}^{2}-2rs_{validation}s_{testing}}}$ **Eq. 7**

## **Association between the classification error of the models trained with structural neuroimaging and demographic, clinical and imaging variables**

The main effect of several demographic, clinical and imaging variables (age at scan, sex, IQ, GAF and CAARMS at baseline, interval between baseline and scan acquisition age, scan acquisition protocol and IQR – independent variables) on the classification error (dependent variable) of each tested classifier was tested using a repeated measures design with generalized estimation equations (GEE). In detail, a GEE model was fit for each effect of interest including the independent variable of interest as a between subjects variable and each tested combination of feature type (i.e., ROIGM, ROIWM, ROISurface, VBGM, or VBWM), feature manipulation (i.e., feature dimensionality reduction – principal component analysis or feature selection – no feature selection or forward feature selection), and CV scheme (i.e., LSO, LPO, or 5 fold CV), and bootstrapped samples (i.e., each of the five samples) as within-subjects variables. GEE was chosen given a) that not all ARMS-NT subjects will be represented in all bootstrapped samples (as opposite to the ARMS-T group, which is the same across bootstrapped samples); and b) its superior management of missing data in repeated measures designs, relative to ANOVA (29). Furthermore, GEE was run with an “Unstructured” covariance matrix between each level of the within-subject factors and a binomial distribution with a logit link was assumed for the classification error. The statistical significance of each effect of interest was tested with a Wald chi squared test and the correspondent *p*-values were corrected for multiple testing (i.e., for 8 statistical tests – every independent variable of interest). The effect was considered statistically significant at a level of significance of 5% (i.e., FDR corrected *p* < .05). Moreover, effect sizes were computed for each effect as the odds ratio (OR), which was computed from the beta (β) parameter estimate from the GEE model (**SEq. 12**) for the independent continuous variable (i.e., age at scan, intelligence quotient, GAF and CAARMS at baseline, interval between baseline and scan ages and weighted average image quality rating) or each level of the independent categorical variables (i.e., sex and scan acquisition protocol).

$OR=e^{\beta}$ **(SEq. 12)**

# **Supplementary results**

## **Image quality control**

Image quality measures, i.e., NCR, ICR, RES, and IQR, are in **STable 8**. A main effect of scan acquisition protocol was found on every measure (*p* < .001). The main effects of transition and interaction effect of protocol by transition were not statistically significant on any of the quality measures. Furthermore, 48 (48.5%) images achieved a good weighted quality (23.3% rated with a B and 25.3% with a B-), 49 (49.5%) achieved a satisfactory weighted quality (24.2% rated with a C+, 16.1% with a C, and 6.1% with a C-), and 2 (2%) achieved a sufficient weighted quality (1% rated with a D+ and 1% with a D). Images with good overall image quality ratings (i.e., rated with B/B-) were acquired mainly with scan acquisition protocol 3, whereas images from protocols 1 and 2 were mainly rated as having a satisfactory overall quality (i.e., rated with C+/C/C-) (**SFigure 2**). All the subjects’ images surpassed the overall weighted quality threshold (i.e., sufficient/D) and, therefore, were included in further analyses.

## **Comparison between training and testing model performance**

The difference between the testing and the validation BAC was statistically significant (for 15 neuroimaging classifiers (out of 24 possible combinations of feature type and manipulation and CV schemes; *p* < .05; **STables 9** and **10**; **SFigures 3-6**) and for the SNPs-based classifiers (*p* < .001; d = 1.2; **STable 11**; **SFigure 7**). The difference was non-significant for all the other comparisons (**STables 11** and **12**; **SFigures 7** and **8**). In cases where the difference was significant, the validation BAC was on average lower than the testing BAC (**SFigures 3-7**; see also **STables 9-11** for effect sizes). Moreover, very large effect sizes (i.e., *d* > 1.2) were observed for 4 classifiers trained with the following combinations: ROIGM, forward feature selection and a) LSO; b) LPO; or c) 5-fold CV; and d) ROISurface, forward feature selection and LPO CV.

## **Association between the classification error and demographic, clinical and imaging variables**

The main effect of scan acquisition protocol (FDR corrected *p* = .016; OR_protocol 1_ = 0.62 and OR_protocol 2_ = 0.69, when compared to protocol 3) and IQR (FDR corrected *p* = .019; OR = 0.72) on the classification error were statistically significant (**STable 13**). Subjects are less likely to be wrongly classified if they are a) scanned using protocol 1 or 2 (instead of protocol 3) and b) if their structural MRI’s weighted average quality rating increases. The main effect of age at scan, sex, IQ, GAF and CAARMS at baseline and interval between baseline and scan age on the classification error were not significant.

# **Supplementary Discussion**

## **Association between the classification error and demographic, clinical and imaging variables**

We also investigated the association between the classification error of models trained with sMRI data and several extraneous variables, namely demographic, clinical and imaging variables. Surprisingly, we found a significant association between scan acquisition protocol and sMRI’s weighted average quality rating and the classification error, albeit they were balanced between the groups (i.e., ARMS-T and ARMS-NT). Indeed, these results show that subjects are more likely to be wrongly classified if they are scanned with the acquisition protocol from the 3T scanner and if their MRI images have a lower weighted average quality rating, i.e., better quality. As a matter of fact, the images acquired with the 3T protocol are the ones showing the highest overall quality from the whole sample, therefore, it is not surprising that both variables’ (i.e., the protocol and average quality) effects are significant. Although I applied a subsampling strategy that keeps the groups balanced within each protocol, maximizing in this way the sample size, this seems to be insufficient to dilute the effect of protocol on the classification accuracy.

# **Supplementary Tables**

**STable 1.** Regions of interest (ROI) for which the grey and white matter volume were extracted. Volumes were extracted for the left and right side of each ROI and using the Hammers atlas.

| Hippocampus | Inferolateral remainder of parietal lobe |
| --- | --- |
| Amygdala | Caudate nucleus |
| Anterior temporal lobe, medial part | Nucleus accumbens |
| Anterior temporal lobe, lateral part | Putamen |
| Parahippocampal and ambient gyri | Thalamus |
| Superior temporal gyrus | Pallidum |
| Middle and inferior temporal gyri | Corpus callosum |
| Fusiform gyrus | Precentral gyrus |
| Cerebellum | Gyrus rectus |
| Brainstem | Orbitofrontal gyri |
| Insula | Inferior frontal gyrus |
| Lateral remainder of occipital lobe | Superior frontal gyrus |
| Gyrus cinguli, anterior part | Postcentral gyrus |
| Gyrus cinguli, posterior part | Superior parietal gyrus |
| Middle frontal gyrus | Lingual gyrus |
| Posterior temporal lobe | Cuneus |

**STable** **2.** Regions of interest (ROI) for which cortical thickness, gyrification index, depth of sulci, and local surface complexity were extracted. These measures were extracted for the left and right side of each ROI and using the Desikan-Killiany atlas.

| Banks of the superior temporal sulcus | Medial orbital frontal cortex | Rostral anterior cingulate cortex |
| --- | --- | --- |
| Caudal anterior-cingulate cortex | Middle temporal gyrus | Rostral middle frontal gyrus |
| Caudal middle frontal gyrus | Parahippocampal gyrus | Superior frontal gyrus |
| Cuneus cortex | Paracentral lobule | Superior parietal cortex |
| Entorhinal cortex | Pars opercularis | Superior temporal gyrus |
| Fusiform gyrus | Pars orbitalis | Supramarginal gyrus |
| Inferior parietal cortex | Pars triangularis | Frontal pole |
| Inferior temporal gyrus | Pericalcarine cortex | Temporal pole |
| Isthmus–cingulate cortex | Postcentral gyrus | Transverse temporal cortex |
| Lateral occipital cortex | Posterior-cingulate cortex | Insula |
| Lateral orbital frontal cortex | Precentral gyrus |  |
| Lingual gyrus | Precuneus cortex |  |

**STable 3.** Number of subjects at an at-risk mental state (ARMS) per self-reported ethnicity and prognosis (i.e. transition to psychosis, ARMS-T, or remission of symptoms, ARMS-NT) with genome-wide genotyped data.

| **Self-reported ethnicity** | **ARMS-T** | **ARMS-NT** |
| --- | --- | --- |
| White (n=77) | 21 | 56 |
| Black (n=41) | 16 | 25 |
| Asian (n=6) | 2 | 4 |
| Mixed (n=9) | 2 | 7 |

**STable 4.** Number of psychosis associated SNPs with a statistical significance level below a given threshold (i.e. p < 10^-2^, p < 10^-3^, p < 10^-4^, or p < 10^-5^) in the original study (21). The number of SNPs from each subset (i.e. defined by each threshold) that are available in the ARMS sample is also represented.

| **#SNPs** | ***p* < 10^-2^** | ***p* < 10^-3^** | ***p* < 10^-4^** | ***p* < 10^-5^** |
| --- | --- | --- | --- | --- |
| (21) | 307 632 | 98 130 | 41 442 | 21 498 |
| ARMS sample | 79 247 | 31 565 | 14 791 | 8 102 |

**STable 5.** Number of principal components extracted from the data in each inner CV cycle in each CV scheme that was used (i.e., leave-one scan protocol-out CV, leave-one per group-out CV, 5-fold CV) and for each feature type (i.e., voxel-based grey (VBGM) or white (VBWM) matter volume maps) for which principal component analysis was used to reduce feature space dimensions. Both the maximum number of components that is possible to extract from data and the average number of components explaining up to 80% of the variance in the data per inner CV across bootstrapped samples that were indeed extracted are shown.

| **Inner CV cycle** | **Principal components to extract**  Maximum  Mean (standard deviation) |
| --- | --- |
| Leave-one scan protocol-out CV scheme |  |
| Inner CV cycle 1: Protocol 1 (n=28) | 14  VBGM: 6.8 (0.4)  VBWM: 6.8 (0.4) |
| Inner CV cycle 2: Protocol 2 (n=6) | 3  VBGM: 1 (0)  VBWM: 1 (0) |
| Inner CV cycle 3: Protocol 3 (n=12) | 6  VBGM: 3 (0)  VBWM: 2.4 (0.5) |
| Leave-one per group-out CV scheme |  |
| Each inner CV cycle (n=42) | 21  VBGM: 9.9 (0.0)  VBWM: 9.6 (0.0) |
| 5-fold CV scheme |  |
| Each inner CV cycle (n=30) | 15  VBGM: 3.6 (0.2)  VBWM: 3.4 (0.4) |

**STable 6.** Number of genes per brain tissue for which an eQTL score was computed.

| **Brain tissue** | **#genes** |
| --- | --- |
| Frontal cortex | 21 |
| Temporal cortex | 17 |
| Occipital cortex | 16 |
| Putamen | 16 |
| Substantia nigra | 13 |
| Hippocampus | 11 |
| Cerebellum | 28 |
| White matter | 19 |

**STable7.** Contribution of each environmental risk factor to the environmental risk score (ERS). RR: relative risk.

| **Risk factor**  (meta-analysis) | **Sub-categories** | **RR from meta-analysis** | **Proportion of population** (%)^1^ | **Scaled log(RR)** | **ERS component** |
| --- | --- | --- | --- | --- | --- |
| Upbringing urbanicity  (30) | Low | 1.16 | 33.3 | -0.14 | -1.5 |
|  | Medium | 1.55 | 33.3 | -0.01 | 0 |
|  | High | 2.07 | 33.3 | 0.11 | 1 |
| Cannabis consumption  (31) | No exposure | 1 | 70 | -0.12 | -1 |
|  | Little/moderate | 1.41 | 15 | 0.02 | 0 |
|  | High exposure | 2.77 | 15 | 0.32 | 3 |
| Paternal age  (32) | <40 | 1 | 92.1 | -0.01 | 0 |
|  | 40-50 | 1.17 | 7.1 | 0.06 | 0.5 |
|  | >50 | 1.60 | 0.8 | 0.19 | 2 |
| Childhood trauma (overall)  (33) | No exposure | 1 | 73 | -0.17 | -1.5 |
|  | Any exposure | 2.78 | 27 | 0.27 | 2,5 |
| Season of birth  (34) | Summer/Autumn | 1 | 96.7 | -1.00x10^-3^ | 0 |
|  | Winter/Spring | 1.07 | 3.3 | 0.03 | 0.5 |
| Tobacco consumption  (35) | No exposure | 1 | 70.6 | -0.11 | -1 |
|  | Any exposure | 1.99 | 29.4 | 0.19 | 2 |
| Ethnic minority  (36) | Native | 1 | 92.4 | -0.04 | -0.5 |
|  | Black | 4 | 1.3 | 0.56 | 5.5 |
|  | White | 1.8 | 2.8 | 0.22 | 2 |
|  | Other | 2 | 3.5 | 0.26 | 2.5 |

^1^Source for season of birth: (34) and for tobacco consumption: WHO – Prevalence of tobacco smoking 2016 ([http://gamapserver.who.int/gho/interactiveharts/tobacco/use/atlas.html](http://gamapserver.who.int/gho/interactive_charts/tobacco/use/atlas.html)).

**STable 8.** Image quality assessment performed in the structural MRI data of the ARMS sample.

|  | Protocol 1 |  | Protocol 2 |  | Protocol 3 |  | Group comparison |
| --- | --- | --- | --- | --- | --- | --- | --- |
|  | ARMS-T  (n = 14) | ARMS-NT  (n = 19) | ARMS-T  (n = 3) | ARMS-NT  (n = 16) | ARMS-T  (n = 6) | ARMS-NT  (n = 41) |  |
| NCR | 3.2 ± 0.4  [2.6 4.0] | 3.2 ± 0.4  [2.8 4.3] | 2.9 ± 0.1  [2.8 3.0] | 3.0 ± 0.2  [2.5 3.3] | 2.2 ± 0.3  [2.0 2.8] | 2.2 ± 0.2  [1.9 2.9] | Protocol: p < .001***  Transition: p = .848  Protocol x Transition: p = .825 |
| ICR | 1.6 ± 0.1  [1.4 1.7] | 1.6 ± 0.1  [1.4 1.8] | 1.7 ± 0.2  [1.6 1.9] | 1.7 ± 0.1  [1.5 1.9] | 2.7 ± 0.4  [2.2 3.5] | 2.4 ± 0.3  [1.9 3.0] | Protocol: p < .001***  Transition: p = .234  Protocol x Transition: p = .113 |
| RES | 2.2 | 2.2 | 2.2 | 2.2 | 2.2 | 2.2 | Protocol: p = < .001***  Transition: p = .146  Protocol x Transition: p = .074 |
| IQR | 2.9 ± 0.3  [2.4 3.7] | 2.9 ± 0.3  [2.6 3.9] | 2.7 ± 0.1  [2.6 2.8] | 2.8 ± 0.2  [2.4 3.1] | 2.2 ± 0.2  [2.1 2.6] | 2.2 ± 0.1  [2.1 2.7] | Protocol: p < .001***  Transition: p = .882  Protocol x Transition: p = .789 |

**Data format:** mean ± standard deviation [min max]. **Abbreviations:** ARMS: at-risk mental state; ARMS-T: individuals at an ARMS who later transitioned to psychosis; ARMS-NT: individuals at an ARMS who did not transitioned to psychosis; ICR: inhomogeneity contrast ratio; IQR: weighted average image quality rating; NCR: noise contrast ratio; RES: root-mean-squared resolution. *p<.05; **p<.01; ***p<.001

**STable 9.** Statistical significance of the difference between the testing (i.e., from the inner CV cycle) and the validation (i.e., from the outer CV cycle) balanced accuracy for each tested combination of regional feature type [i.e., regional-based grey (ROIGM) or white (ROIWM) matter volume; or surface-based regional cortical thickness, gyrification, sulci and complexity indexes (ROISurface)], feature selection [i.e., no feature selection (NoFS) or forward feature selection (FFS)] and cross-validation (CV) scheme [i.e., leave-one scan acquisition protocol-out (LSO) CV; leave-one per group-out (LPO) CV; or 5-fold CV]. The difference between the testing and validation BAC was considered statistically significant at a level of 5% (i.e., p < .05). Effects sizes were computed as the Cohen’s d between validation and testing balanced accuracies.

|  | **ROIGM** | | **ROIWM** | | **ROISurface** | |
| --- | --- | --- | --- | --- | --- | --- |
|  | *p* | *d* | *p* | *d* | *p* | *d* |
| **LSO CV scheme** |  |  |  |  |  |  |
| **NoFS** | .296 | 0.10 | .002** | -0.21 | .052 | -0.44 |
| **FFS** | .001*** | -1.34 | .019* | -0.19 | .007** | -0.59 |
| **LPO CV scheme** |  |  |  |  |  |  |
| **NoFS** | .903 | -0.11 | < .001*** | -0.21 | .095 | -0.34 |
| **FFS** | < .001*** | -1.50 | .043* | -0.04 | < .001*** | -1.27 |
| **5-fold CV scheme** |  |  |  |  |  |  |
| **NoFS** | .552 | -0.15 | .003** | -0.16 | .936 | 0.03 |
| **FFS** | < .001*** | -1.44 | .110 | -0.16 | < .001*** | -1.00 |

*p < .05, **p < .01, ***p < .001.

**STable 10.** Statistical significance of the difference between the testing (i.e., from the inner CV cycle) and the validation (i.e., from the outer CV cycle) balanced accuracy for each tested combination of voxel-wise feature type [i.e., voxel-based grey (VBGM) or white (VBWM) matter volume maps], principal component analysis and cross-validation (CV) scheme [i.e., leave-one scan acquisition protocol-out (LSO) CV; leave-one per group-out (LPO) CV; or 5-fold CV]. The difference between the testing and validation BAC was considered statistically significant at a level of 5% (i.e., p < .05). Effects sizes were computed as the Cohen’s d between validation and testing balanced accuracies.

|  | **VBGM** | | **VBWM** | |
| --- | --- | --- | --- | --- |
|  | *p* | *d* | *p* | *d* |
| **LSO CV scheme** | .198 | -0.45 | .004** | -0.12 |
| **LPO CV scheme** | .018* | -0.53 | .267 | -0.25 |
| **5-fold CV scheme** | .004** | -0.13 | .050* | -0.43 |

*p < .05, **p < .01.

**STable 11.** Statistical significance of the difference between the testing (i.e., from the inner CV cycle) and the validation (i.e., from the outer CV cycle) balanced accuracy for models trained with genetic (single nucleotide polymorphisms (SNPs) and expression quantitate trait loci (eQTL) scores) and environmental (environmental risk factors) features. The difference between the testing and validation BAC was considered statistically significant at a level of 5% (i.e., p < .05). Effects sizes were computed as the Cohen’s d between validation and testing balanced accuracies.

|  | *p* | *d* |
| --- | --- | --- |
| **SNPs** | .028* | 1.08 |
| **eQTL scores** | .063 | 1.60 |
| **Environmental risk factors** | .063 | 2.91 |

*p < .05.

**STable 12.** Statistical significance of the difference between the training and the testing balanced accuracy for the models trained with polygenic risk score (PRS) and the environmental risk score (ERS). The difference between the training and testing BAC was considered statistically significant at a level of 5% (i.e., p < .05). Effects sizes were computed as the Cohen’s d between testing and training balanced accuracies.

|  | *p* | *d* |
| --- | --- | --- |
| **PRS** | .125 | 0.79 |
| **ERS** | .181 | 0.91 |

**STable 13.** Main effect of demographic, clinical and imaging variables on the classification error of each tested classifier. The effect was considered statistically significant at a significance level of 5% (i.e., FDR corrected p value < .05). Effects sizes were computed for each effect as the odds ratio (OR) for the continuous variables (i.e., age at scan, intelligence quotient, GAF and CAARMS at baseline, interval between baseline and scan ages and weighted average image quality rating) and each level of the categorical variables (i.e., sex, handedness, self-reported ethnicity, and scan acquisition protocol).

|  | **Wald χ^2^**(df,n) | ***p*** | **FDR *p*** | **Effect size**  OR; 95% CI |
| --- | --- | --- | --- | --- |
| **Age at scan** | χ^2^(1,5520) = 1.41 | .235 | .471 | 0.99; [1.97 1.01] |
| **Sex** | χ^2^(1,5520) = 0.32 | .569 | .608 | Male: 1.00  Female: 1.05 [0.88 1.26] |
| **Intelligence quotient** | χ^2^(1,5520) = 0.26 | .608 | .608 | 0.98 [0.90 1.07] |
| **GAF at baseline** | χ^2^(1,5520) = 0.46 | .499 | .608 | 1.00 [0.99 1.01] |
| **CAARMS at baseline** | χ^2^(1,5478) = 2.21 | .138 | .367 | 1.00 [1.00 1.01] |
| **Interval between baseline and scan age** | χ^2^(1,5520) = 0.97 | .326 | .521 | 0.96 [0.89 1.04] |
| **Scan acquisition protocol** | χ^2^(2,5520) = 12.46 | .002** | .016* | Protocol 1 = 0.62 [0.48 0.81]  Protocol 2 = 0.69 [0.51 0.93]  Protocol 3 = 1.00 |
| **Weighted average image quality rating** | χ^2^(1,5520) = 8.02 | .005** | .019* | 0.72 [0.58 0.91] |

*p<.05

# **Supplementary Figures**


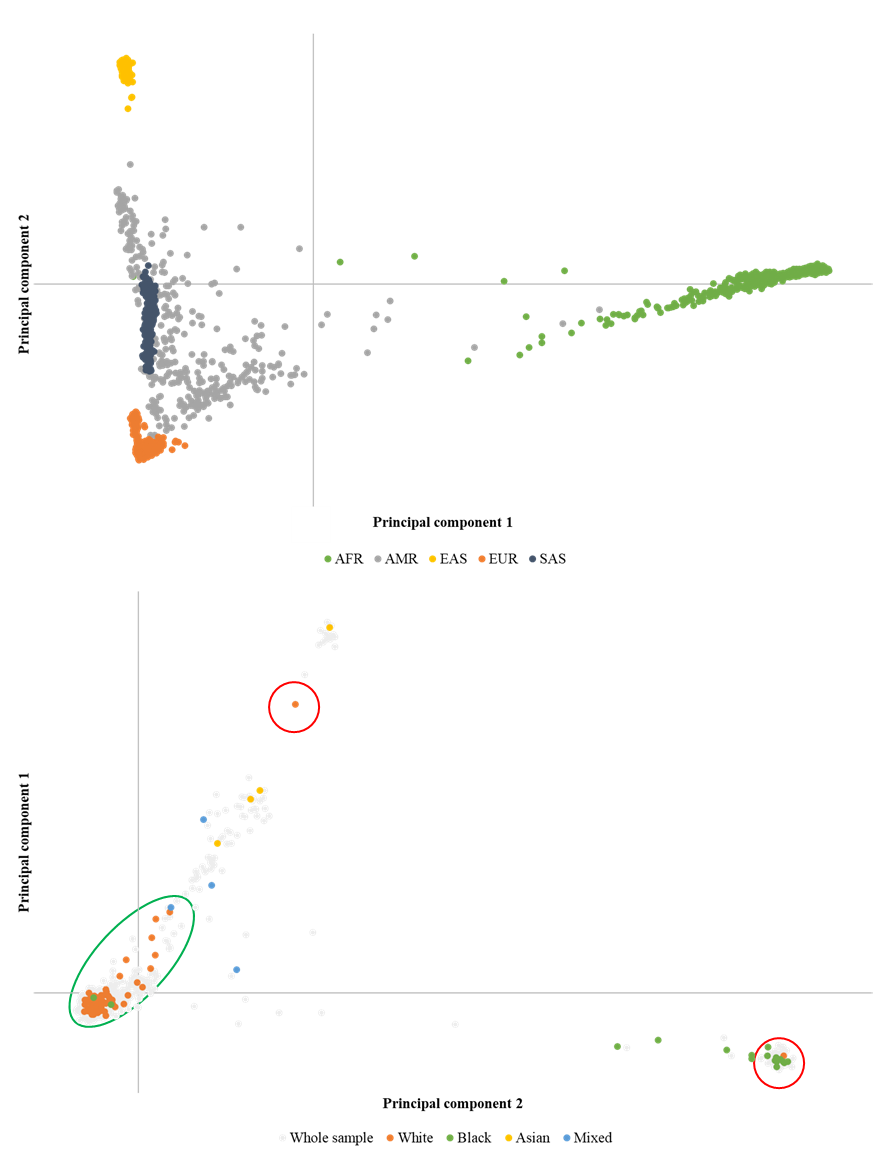


**SFigure 1.** Population stratification analysis from the reference dataset (i.e. 1000 Genomes; top) and the study sample (i.e. ARMS sample, bottom). ARMS subjects selected for further analysis met the following two criteria: a) self-reported as being ‘white’ (orange dots in the bottom plot); and b) show a genetic structure similar to the that of the reference dataset’s subjects with an European ancestry (orange dots in the top plot). Therefore, the ARMS subjects included (i.e. 75 subjects) in the final sample are highlighted by the green ancestry (i.e. met the two criteria) and the ones excluded (i.e. 2 subjects) are highlighted by the red circles (i.e. they have reported as being white, but showed a genetic structure similar to other populations – Asian and African. AFR: African, AS: Asian, AMR: American, EAS: East Asian, EUR: European, SAS: South Asian ancestries.


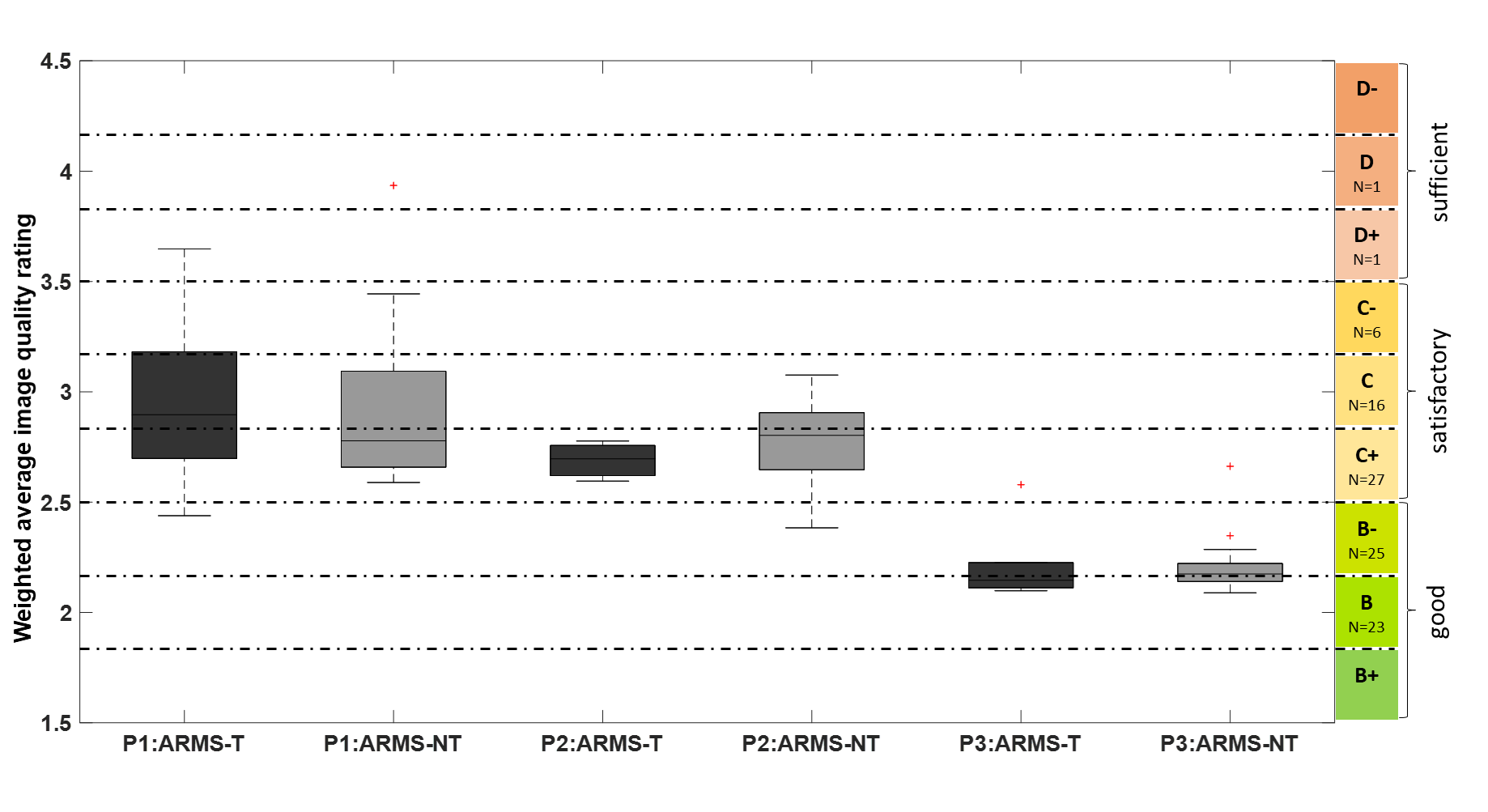


**SFigure 2****.** Weighted average image quality rating computed by the quality ascertainment framework of CAT12 using the noise (i.e., noise contrast ratio) and bias (i.e., inhomogeneity contrast ratio) information of each scan protocol [i.e., scan acquisition protocol 1 (P1), 2 (P2), or 3 (P3)] and for each group [i.e., individuals at an at-risk mental state who transitioned to psychosis (ARMS-T) or who did not (ARMS-NT)]. The quality ascertainment framework maps the rating scores to image quality grades (A-F) shown on the right side of the figure.


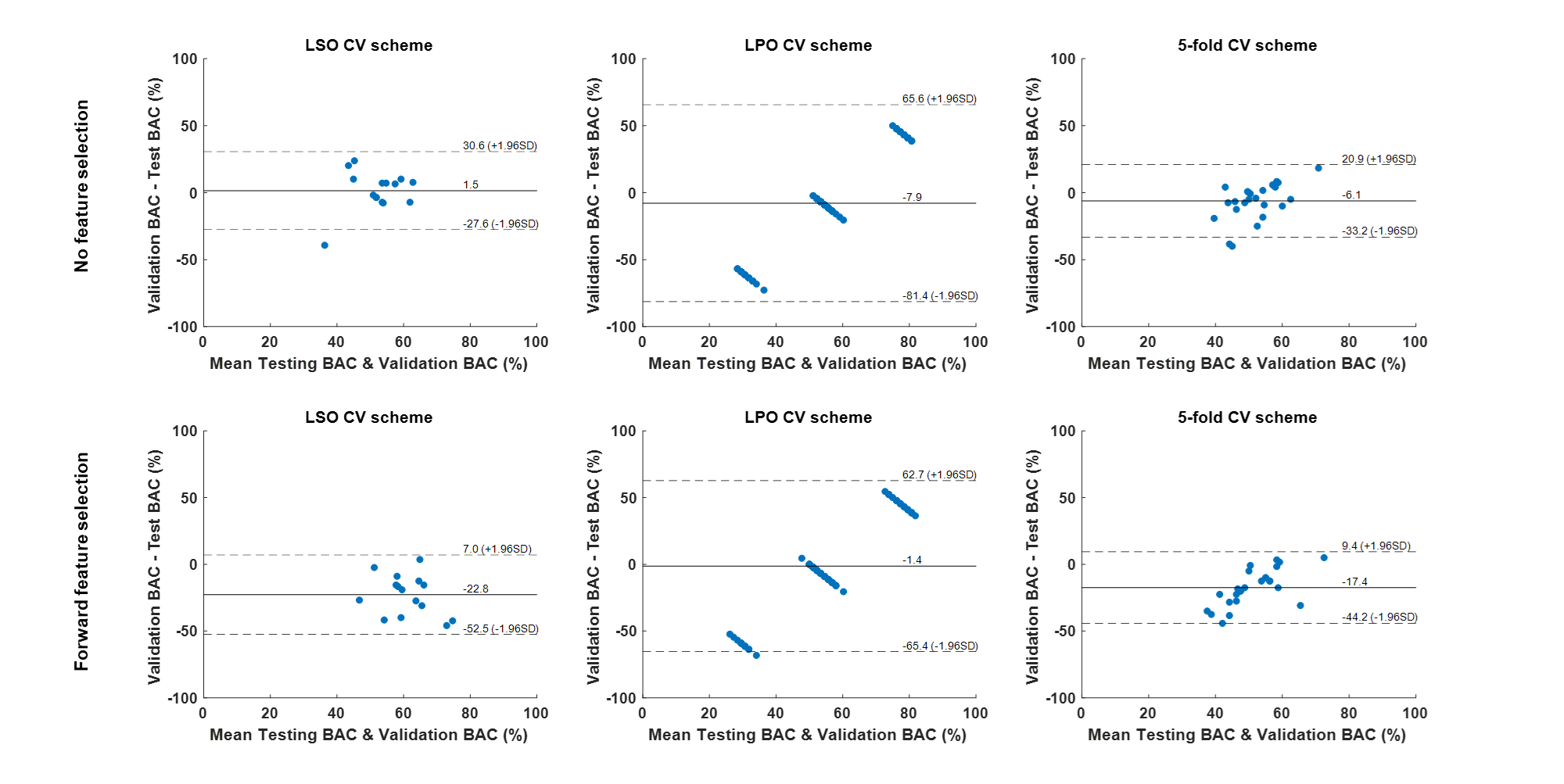


**SFigure 3.** Bland-Altman plot with limits (dashed lines) of agreement for mean (continuous line) testing and validation balanced accuracies (BAC) of classification models trained with regional-based grey matter volumes in combination with feature selection [i.e., no feature selection (top); or forward feature selection (bottom)] and cross-validation (CV) scheme [i.e., leave-one scan acquisition protocol-out (LSO) CV; leave-one per group-out (LPO) CV; and 5-fold CV].


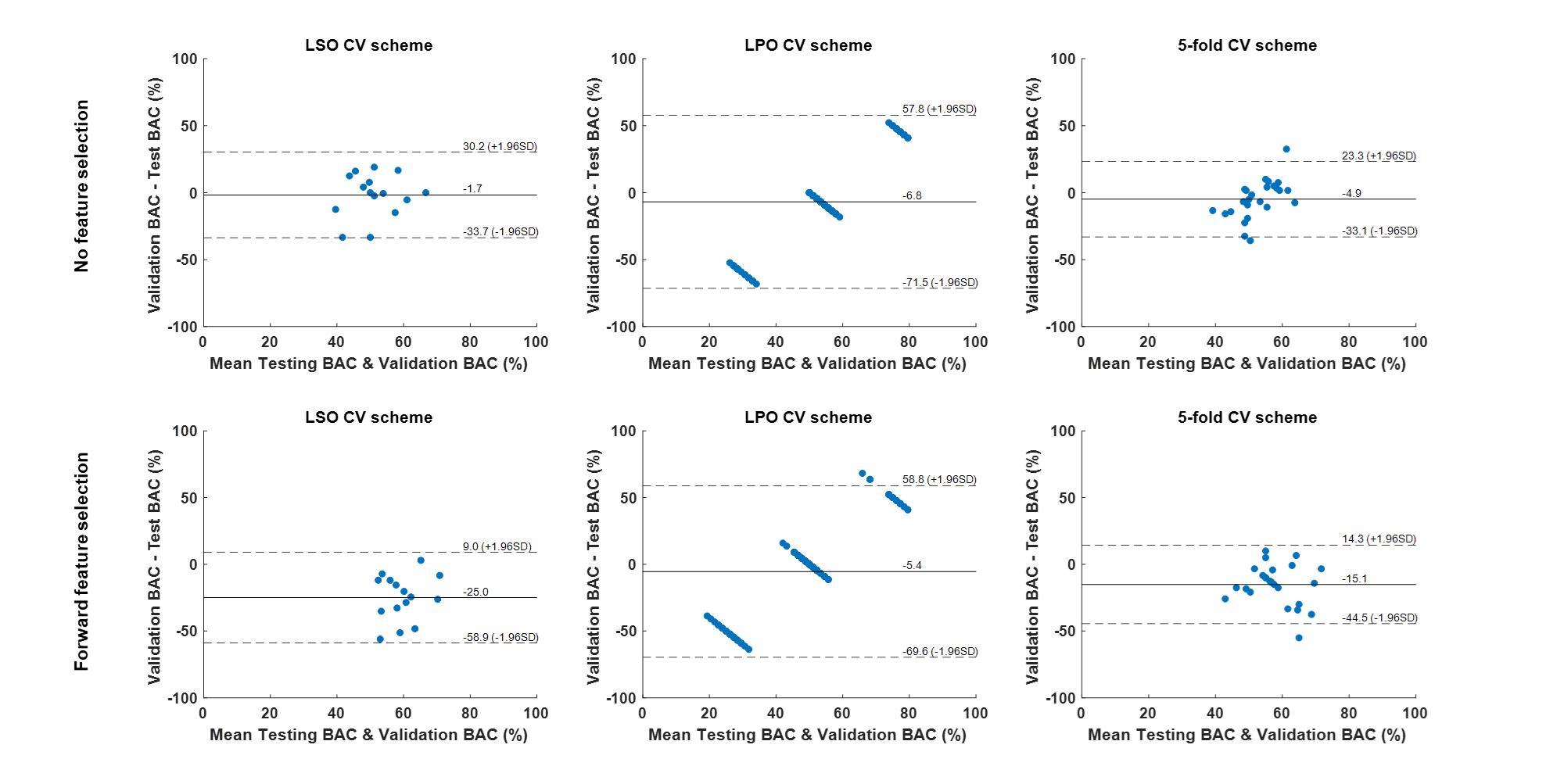


**SFigure 4.** Bland-Altman plot with limits (dashed lines) of agreement for mean (continuous line) testing and validation balanced accuracies (BAC) of classification models trained with regional-based white matter volumes in combination with feature selection [i.e., no feature selection (top); or forward feature selection (bottom)] and cross-validation (CV) scheme [i.e., leave-one scan acquisition protocol-out (LSO) CV; leave-one per group-out (LPO) CV; and 5-fold CV].


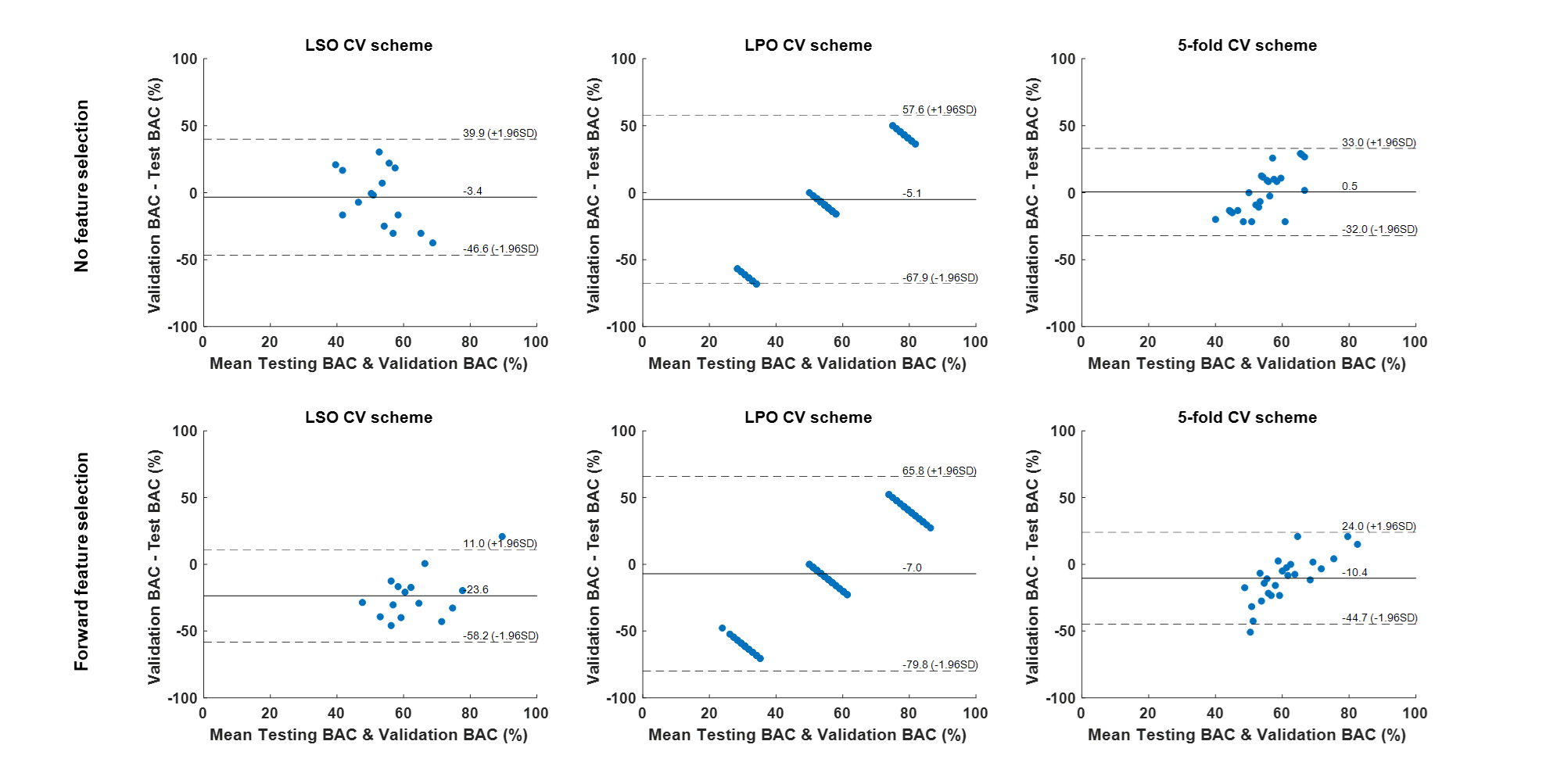


**SFigure 5.** Bland-Altman plot with limits (dashed lines) of agreement for mean (continuous line) testing and validation balanced accuracies (BAC) of classification models trained with surface-based regional cortical thickness, gyrification, sulci and complexity indexes in combination with feature selection [i.e., no feature selection (top); or forward feature selection (bottom)] and cross-validation (CV) scheme [i.e., leave-one scan acquisition protocol-out (LSO) CV; leave-one per group-out (LPO) CV; and 5-fold CV].


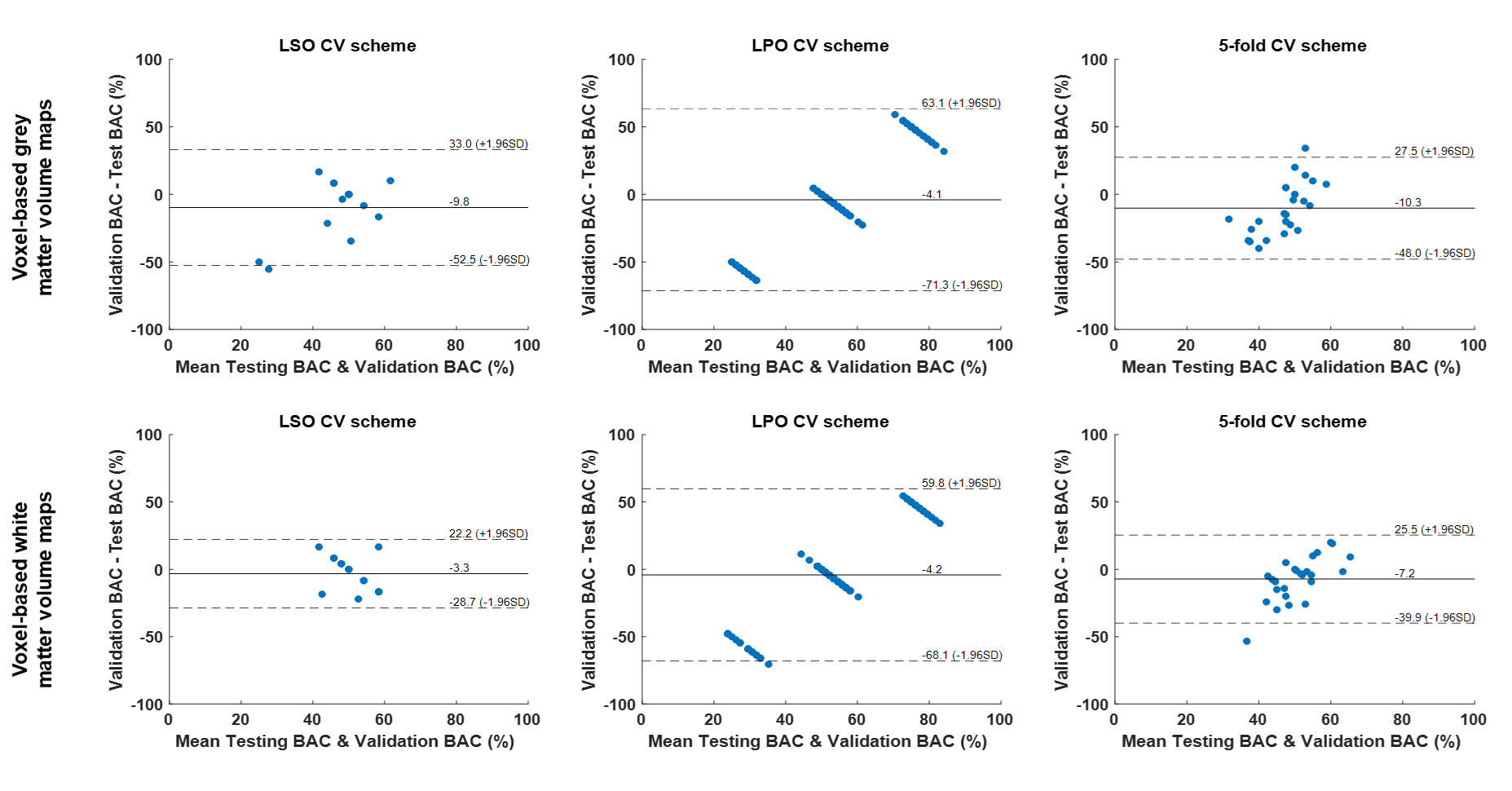


**SFigure 6.** Bland-Altman plot with limits (dashed lines) of agreement for mean (continuous line) testing and validation balanced accuracies (BAC) of classification models trained with voxel-based grey (top) or white (bottom) matter volume maps in combination feature dimensionality reduction through principal component analysis with cross-validation (CV) scheme [i.e., leave-one scan acquisition protocol-out (LSO) CV; leave-one per group-out (LPO) CV; and 5-fold CV].


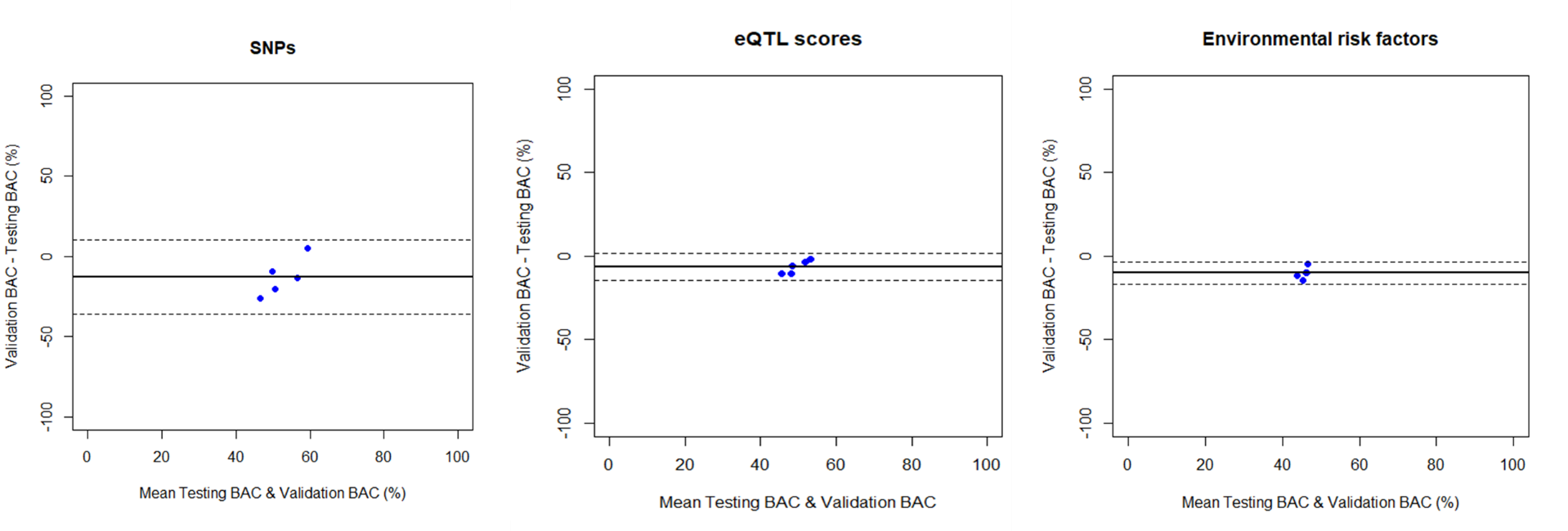


**SFigure 7**. Bland-Altman plot with limits (dashed lines) of agreement for mean (continuous line) testing and validation balanced accuracies (BAC) of classification models trained with the single nucleotide polymorphyms, eQTL scores for psychosis-associated genes expressed in the brain, and for the environmental risk scores.


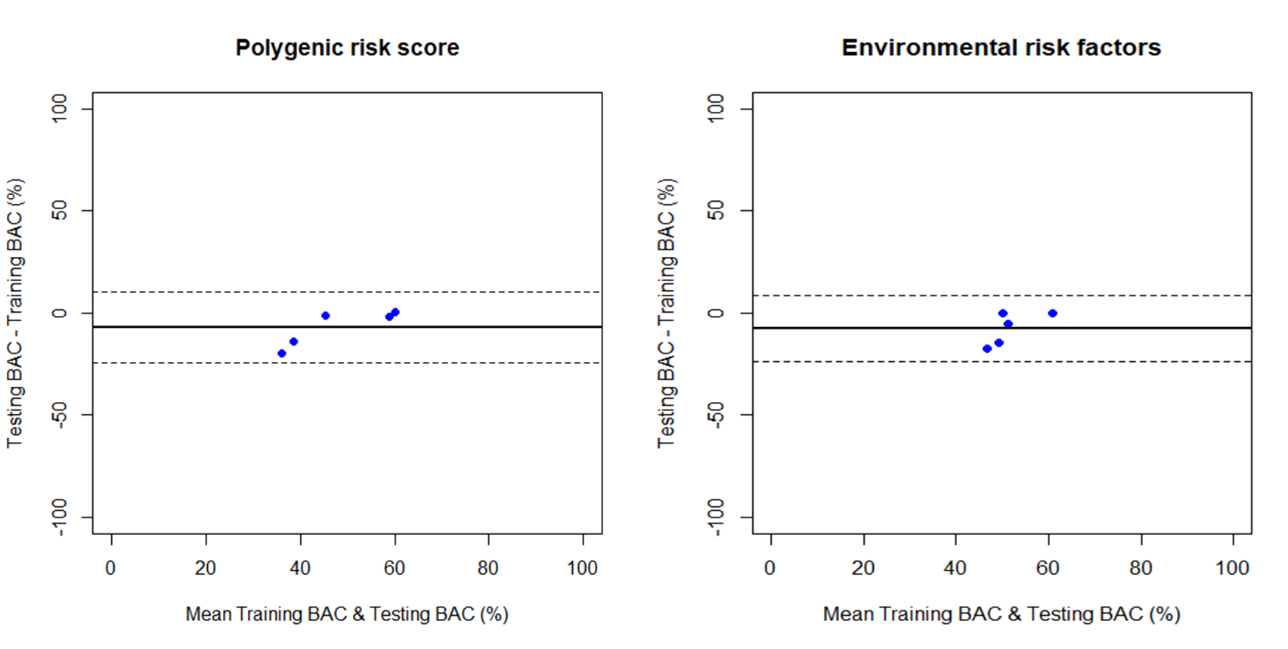


**SFigure 8**. Bland-Altman plot with limits (dashed lines) of agreement for mean (continuous line) training and testing balanced accuracies (BAC) of classification models trained with the polygenic risk score and the environmental risk score.

# **References**

1. Broome MR, Woolley JB, Johns LC, Valmaggia LR, Tabraham P, Gafoor R, et al. Outreach and support in south London (OASIS): implementation of a clinical service for prodromal psychosis and the at risk mental state. Eur Psychiatry. 2005 Aug;20(5):372–8.

2. Phillips LJ, Yung AR, McGorry PD. Identification of young people at risk of psychosis: validation of Personal Assessment and Crisis Evaluation Clinic intake criteria. Aust N Z J Psychiatry. 2000 Nov;34 Suppl:S164-9.

3. Nurnberger Jr JI, Blehar MC, Kaufmann CA, York-Cooler C, Simpson SG, Harkavy-Friedman J, et al. Diagnostic Interview for Genetic Studies: Rationale, Unique Features, and Training. Arch Gen Psychiatry. 1994 Nov;51(11):849–59.

4. American Psychiatric Association. Diagnostic and Statistical Manual of Mental Disorders, Fourth Edition, Text Revision (DSM-IV-TR). Vol. 1. Arlington, VA: American Psychiatric Association; 2000.

5. Nelson HE. The National Adult Reading Test (NART): Test Manual. Wind UK NFER-Nelson. 1982;124(3):0–25.

6. Tavares V, Prata D, Ferreira HA. Comparing SPM12 and CAT12 segmentation pipelines: a brain tissue volume-based age and Alzheimer’s disease study. J Neurosci Methods. 2020 Mar;334:108565.

7. Ashburner J. A fast diffeomorphic image registration algorithm. Neuroimage. 2007;38(1):95–113.

8. Hammers A, Allom R, Koepp MJ, Free SL, Myers R, Lemieux L, et al. Three-dimensional maximum probability atlas of the human brain, with particular reference to the temporal lobe. Hum Brain Mapp. 2003;19(4):224–47.

9. Luders E, Thompson PM, Narr KL, Toga AW, Jancke L, Gaser C. A curvature-based approach to estimate local gyrification on the cortical surface. Neuroimage. 2006;29(4):1224–30.

10. Yotter RA, Nenadic I, Ziegler G, Thompson PM, Gaser C. Local cortical surface complexity maps from spherical harmonic reconstructions. Neuroimage. 2011;56(3):961–73.

11. Desikan RS, Ségonne F, Fischl B, Quinn BT, Dickerson BC, Blacker D, et al. An automated labeling system for subdividing the human cerebral cortex on MRI scans into gyral based regions of interest. Neuroimage. 2006;31(3):968–80.

12. Dahnke R, Ziegler G, Grosskreutz J, Gaser C. Retrospective Quality Assurance of MR Images. 2013.

13. Vassos E, Di Forti M, Coleman J, Iyegbe C, Prata D, Euesden J, et al. An Examination of Polygenic Score Risk Prediction in Individuals With First-Episode Psychosis. Biol Psychiatry. 2017 Mar;81(6):470–7.

14. Bramon E, Pirinen M, Strange A, Lin K, Freeman C, Bellenguez C, et al. A genome-wide association analysis of a broad psychosis phenotype identifies three loci for further investigation. Biol Psychiatry. 2014;75(5):386–97.

15. Howie BN, Donnelly P, Marchini J. A Flexible and Accurate Genotype Imputation Method for the Next Generation of Genome-Wide Association Studies. Schork NJ, editor. PLoS Genet. 2009 Jun;5(6):e1000529.

16. Auton A, Abecasis GR, Altshuler DM, Durbin RM, Bentley DR, Chakravarti A, et al. A global reference for human genetic variation. Nature. 2015;526(7571):68–74.

17. Marees AT, de Kluiver H, Stringer S, Vorspan F, Curis E, Marie-Claire C, et al. A tutorial on conducting genome-wide association studies: Quality control and statistical analysis. Int J Methods Psychiatr Res. 2018 Jun;27(2):e1608.

18. Chang CC, Chow CC, Tellier LCAM, Vattikuti S, Purcell SM, Lee JJ. Second-generation PLINK: Rising to the challenge of larger and richer datasets. Gigascience. 2015;4(1):1–16.

19. Ripke S, Neale BM, Corvin A, Walters JTR, Farh KH, Holmans PA, et al. Biological insights from 108 schizophrenia-associated genetic loci. Nature. 2014;511(7510):421–7.

20. Vassos E, Di Forti M, Coleman J, Iyegbe C, Prata D, Euesden J, et al. An Examination of Polygenic Score Risk Prediction in Individuals with First Episode Psychosis. Biol Psychiatry. 2016;0(0):135–45.

21. Pardiñas AF, Holmans P, Pocklington AJ, Escott-Price V, Ripke S, Carrera N, et al. Common schizophrenia alleles are enriched in mutation-intolerant genes and in regions under strong background selection. Nat Genet. 2018;50(3):381–9.

22. Tavares V, Monteiro J, Vassos E, Coleman J, Prata D. Evaluation of Genotype-Based Gene Expression Model Performance: A Cross-Framework and Cross-Dataset Study. Genes (Basel). 2021 Sep;12(10):1531.

23. Ramasamy A, Trabzuni D, Guelfi S, Varghese V, Smith C, Walker R, et al. Genetic variability in the regulation of gene expression in ten regions of the human brain. Nat Neurosci. 2014;17(10):1418–28.

24. Vassos E, Sham P, Kempton M, Trotta A, Stilo SA, Gayer-Anderson C, et al. The Maudsley environmental risk score for psychosis. Psychol Med. 2020 Sep;50(13):2213–20.

25. Bifulco A, Bernazzani O, Moran PM, Jacobs C. The childhood experience of care and abuse questionnaire (CECA.Q): Validation in a community series. Br J Clin Psychol. 2005 Nov;44(4):563–81.

26. Hubert M, Rousseeuw P, Verdonck T. Robust PCA for skewed data and its outlier map. Comput Stat Data Anal. 2009 Apr;53(6):2264–74.

27. Hubert M, Rousseeuw PJ, Vanden Branden K. ROBPCA: A new approach to robust principal component analysis. Technometrics. 2005;47(1):64–79.

28. Zou H, Hastie T. Regularization and variable selection via the elastic net. J R Stat Soc Ser B (Statistical Methodol. 2005 Apr;67(2):301–20.

29. Hubbard AE, Ahern J, Fleischer NL, Laan M Van Der, Lippman SA, Jewell N, et al. To GEE or not to GEE: Comparing population average and mixed models for estimating the associations between neighborhood risk factors and health. Epidemiology. 2010;21(4):467–74.

30. Vassos E, Pedersen CB, Murray RM, Collier DA, Lewis CM. Meta-Analysis of the Association of Urbanicity With Schizophrenia. Schizophr Bull. 2012 Nov;38(6):1118–23.

31. Marconi A, Di Forti M, Lewis CM, Murray RM, Vassos E. Meta-Analysis of the association between the level of cannabis use and risk of psychosis. Schizophr Bull. 2016;42(5):1262–9.

32. Miller B, Messias E, Miettunen J, Alaräisänen A, Järvelin MR, Koponen H, et al. Meta-analysis of paternal age and schizophrenia risk in male versus female offspring. Schizophr Bull. 2011;37(5):1039–47.

33. Varese F, Smeets F, Drukker M, Lieverse R, Lataster T, Viechtbauer W, et al. Childhood adversities increase the risk of psychosis: A meta-analysis of patient-control, prospective-and cross-sectional cohort studies. Schizophr Bull. 2012;38(4):661–71.

34. Davies GJ, Welham J, Torrey EF, McGrath J. Season of birth effect and latitude: A systematic review and meta-analysis of Northern hemisphere schizophrenia studies. Schizophr Res. 2003;41(1):62.

35. Hunter A, Murray R, Asher L, Leonardi-Bee J. The Effects of Tobacco Smoking, and Prenatal Tobacco Smoke Exposure, on Risk of Schizophrenia: A Systematic Review and Meta-Analysis. Nicotine Tob Res. 2018;(August):1–8.

36. Bourque F, Van Der Ven E, Malla A. A meta-analysis of the risk for psychotic disorders among first- and second-generation immigrants. Psychol Med. 2011;41(5):897–910.
